# Computing by modulating spontaneous cortical activity patterns as a mechanism of active visual processing

**DOI:** 10.1038/s41467-019-12918-8

**Published:** 2019-10-29

**Authors:** Guozhang Chen, Pulin Gong

**Affiliations:** 1 School of Physics, University of Sydney, Sydney, New South Wales 2006 Australia; 2 ARC Center of Excellence for Integrative Brain Function, University of Sydney, Sydney, New South Wales 2006 Australia

**Keywords:** Computational neuroscience, Computational neuroscience, Neural circuits, Sensory processing

## Abstract

Cortical populations produce complex spatiotemporal activity spontaneously without sensory inputs. However, the fundamental computational roles of such spontaneous activity remain unclear. Here, we propose a new neural computation mechanism for understanding how spontaneous activity is actively involved in cortical processing: Computing by Modulating Spontaneous Activity (CMSA). Using biophysically plausible circuit models, we demonstrate that spontaneous activity patterns with dynamical properties, as found in empirical observations, are modulated or redistributed by external stimuli to give rise to neural responses. We find that this CMSA mechanism of generating neural responses provides profound computational advantages, such as actively speeding up cortical processing. We further reveal that the CMSA mechanism provides a unifying explanation for many experimental findings at both the single-neuron and circuit levels, and that CMSA in response to natural stimuli such as face images is the underlying neurophysiological mechanism of perceptual “bubbles” as found in psychophysical studies.

## Abstract (web summary)

The brain’s cortex shows complex activity patterns in the absence of sensory inputs. Here, using computational modelling, the authors demonstrate that cortical spontaneous activity is modulated by sensory input and that this modulation process underlies active visual processing.

## Introduction

Spontaneous neural activity in the absence of stimuli or task performance is widespread in the cortex^1–4^. Understanding the spatiotemporal organization properties and functional roles of such spontaneous activity is of long-standing interest in systems and computational neuroscience^1–10^. Experimental studies with different recording techniques from fMRI BOLD and optical imaging to electrical recordings have increasingly demonstrated that spontaneous intrinsic neural activity is not independent random noise, but is correlated between related neurons^5^, cortical columns^6,7^ and within widely distributed neuroanatomical systems^8,9^. These correlated spontaneous activities give rise to spatially co-activated patterns that have been found at the mesoscopic^10^ and the whole-brain scales^11^, and exhibit wave-like propagation properties^12,13^.

Such spontaneous activity patterns do not disappear in the presence of stimuli or during task performance. Instead, they continue to regulate responses of cortical neurons to sensory inputs. Imaging studies at the whole-brain level have shown that coherent fluctuations of spontaneous activity can account for BOLD response variability and fluctuations in human behavior^14^. At the level of individual neurons, intracellular recordings have shown that spontaneous fluctuations of membrane potentials are related to the trial-by-trial variability of firing rates and response latencies of individual neurons responding to external inputs^5^. These empirical observations indicate that spontaneous activity plays an important role in cortical function. However, the fundamental questions regarding the dynamical nature and the circuit mechanisms underlying the functional role of spontaneous activity remain unclear.

To address these questions, here we propose a novel neural computational mechanism based on dynamical modulation processes of spontaneous activity patterns by external stimuli, which we term Computing by Modulating Spontaneous Activity (CMSA). Our CMSA mechanism can account for why and how spontaneous activity is related to stimuli-evoked neural responses as found at different neural levels in visual cortex^2,5,10^ and ultimately to visual perception^15^. We find that the effect of this computational mechanism is maximal when cortical circuits are in the critical transition of activity states; coherent activity patterns with critical dynamics can quantitatively explain dynamical properties of spontaneous activity in neural circuits, including the co-activation^10^ and wave-like propagation properties^16^, and the spatial structure of neural correlations^7^. Our CMSA mechanism thus reveals the importance of criticality in brain function^17,18^ from a novel perspective. We elucidate the CMSA mechanism using biophysically-based cortical circuit models of spiking neurons and further validate it in firing rate models. These results suggest that the CMSA might be a general computational mechanism of cortical circuits.

## Results

### Critical pattern dynamics of spontaneous activity

**Fig. 1:**
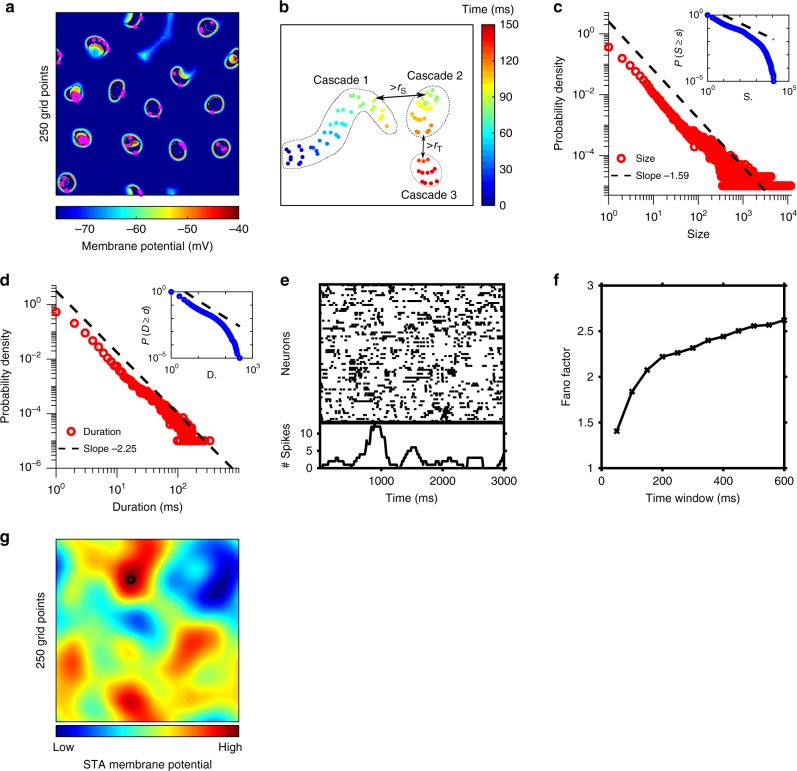
The spatially extended, conductance-based spiking neural network generates co-activated patterns with critical dynamics. **a** The snapshot of spontaneous activity shows co-activated patterns in our circuit model ($250\times 250$ neurons). The magenta dots represent spikes at this moment, illustrating that the fraction of individual neurons participating in a wave is low ($2\pm 0.4$%, mean$\pm$s.d., s.d. represents standard deviation). As these localized activity patterns move in a complex way, all areas of the circuit would be visited by them. **b** An illustrative example of three cascades (dots represent spikes and the color encodes time). For each time step, connected components of spikes are clustered within a radius ${r}_{{\rm{S}}}$. A cascade is defined as a set of clustered spike objects whose center of mass changes by less than ${r}_{{\rm{T}}}$ between successive time (${T}_{2}-{T}_{1}\,=\,1\, {\mathrm{ms}}$). The interaction between cascade 1 and 2 destroys cascade 1. **c** Distribution of cascade sizes follows a power-law function, $P(S) \sim {S}^{-\tau }$. Inset: the complementary cumulative distribution function $P(S\ge s)$ of the same data also follows a power-law function. **d** Same as in **c** but for cascade durations, $P(D) \sim {D}^{-\alpha }$. **e** Top: raster plot shows the spike times of a sub-population of randomly selected 70 excitatory neurons in 3 s. Bottom: single-neuron spike count of a randomly chosen neuron (250 ms bin, sliding over in 10-ms step). **f** Fano factor of spontaneous activity as a function of time window $\Delta t$ during the spontaneous activity and after stimulation. **g** Spike-triggered averaged (STA) membrane potential (${V}_{{\rm{m}}}$) during 2 s shows multiple correlated patterns. The black circle labels the seed neuron. Source data are provided as a Source Data file

We consider a spatially extended, conductance-based spiking circuit model with excitatory and inhibitory neurons that captures the known anatomy and physiology of cortical circuit (see Methods section). It incorporates distance-dependent synaptic connectivity^19,20^, and correlated excitatory and inhibitory synaptic inputs that fluctuate together, as found in intracellular recordings^21^. In the absence of stimuli, the network exhibits multiple co-activated patterns with complex propagating dynamics as shown in Fig. 1a. In this study, we explore the dynamical regime of these patterns, i.e., propagating patterns with critical dynamics, to demonstrate that they provide a mechanistic account of a great variety of dynamical properties of spontaneous coherent activity^1,2,7,10^. Further, we reveal a new computational mechanism of these critical activity patterns underlying cortical processing, thus accounting for the fundamental relationships between spontaneous activity and evoked responses^2,5^.

In this critical regime, the spatially extended network exhibits crescent-shaped waves that move rapidly, and localized, patchy patterns that slowly wander around (Fig. 1a, Supplementary Movie 1); the initial sites and subsequent trajectories of these patterns are seemingly random. If the network is shifted away from this regime by either increasing or decreasing the excitatory coupling strength, the network exhibits regular propagating waves or local patchy patterns within which spikes have randomly wandering motion, respectively; this dynamical regime is thus in the critical transition between these two distinct ordered and disordered activity states (Supplementary Fig. 1, Supplementary Movies 2 and 3). The propagation of the local patterns gives rise to the spatiotemporally contiguous clusters resembling cascades found over many cortical regions in mice^22^. A cascade is initiated from at least one spike; the location of its initiation is seemingly random. In our model, there are multiple, coexisting cascades. In most cases, a cascade terminates due to interactions with other cascades; for instance, the interaction between cascade 1 and 2 results in the termination of cascade 1 (Fig. 1b). As in ref. ^22^, we track these cascades (Fig. 1b) and analyze their size and duration distribution to quantify their critical dynamics (see Methods section). Via maximum likelihood methods^23^, these distributions can be fitted as power-law functions with exponents of $\tau =1.59\pm 0.02$ (Fig. 1c) and $\alpha =2.25\pm 0.01$ (Fig. 1d), respectively. Both power-law fittings apply only for values greater than 2, estimated by the Kolmogorov-Smirnov test (KS, see Methods section). We further compare log-likelihood ratios between these fitted power-law distributions and other distributions including normal, log-normal, gamma and exponential distributions and find that all the log-likelihood ratios are sufficiently positive for both the size and the duration, indicating that most likely the distributions follow power laws ($p\, < \, {1}{0}^{-15}$, Vuong test). The exponent $\tau$ of the power-law size distributions in our model is close to the theoretical value of $\tau =1.5$, and is within the range as found in experimental studies^22^; the value of $\alpha$ is close to the theoretical value $\alpha =2$ and within the range found experimentally^18^. However, if the network is shifted away from the critical transition regime, power laws cease to be the best fitted distributions for both the size and duration distributions (Supplementary Fig. 2), as quantified by the KS test^23^.

Furthermore, we calculate the dynamic range (see Methods section), which is the range of stimulus intensities that can be processed by a neural network^24,25^, and find that the range is maximized in the transition regime (Supplementary Fig. 3), indicating the presence of critical dynamics^24–26^. In addition, it is interesting to note that the transition from the state of the multiple activity patterns with seemingly random propagation to the state with directed propagation is highly analogous to the critical transition underlying the emergence of coherent collection motion in other complex systems, such as flocks of birds^27^, swarms of bacteria^28^, and interacting motile colloids^29^. As in these studies, we introduce propagating velocity fluctuations of activity patterns and calculate the correlation length of these fluctuations (Supplementary Fig. 4a, see Methods section); the correlation length is maximal near the transition regime of our model (Supplementary Fig. 4b). In the transition regime, we further calculate the correlation lengths in our network with different sizes; we find that such spatial correlation lengths do not have a constant value, but scale with the linear size of the network (Supplementary Fig. 4c), which is a signature of criticality^27^. These results thus suggest that the activity patterns near the transition regime exhibit critical dynamics.

We next illustrate that the model in the critical regime can capture key neural dynamics at different neural levels. At the individual-neuron level, there are dynamical transitions between low-activity and high-activity states, corresponding to the fluctuations of firing rates (Fig. 1e, Supplementary Fig. 5). As shown in Fig. 1e, these transitions appear to coexist with variable spike timing of individual neurons, which is a characteristic feature of a doubly stochastic process, as found in experiments^30,31^. To quantify such fluctuations of neural firing activity, we calculate the Fano factor of spike count as a function of the time window $\Delta t$, and find that Fano factor increases as $\Delta t$ increases (Fig. 1f), as found in ref. ^32^. At the circuit level, these patterns generate coherent activity with a spatial structure as found in large-scale neural recordings^1,7,10^. To illustrate this, we first use the spike-triggered average of ${V}_{{\mathrm{m}}}$ (STA-${V}_{{\mathrm{m}}}$) of a seed neuron (see Methods section), and find multiple patches that are highly correlated to the spikes of the seed neuron (Fig. 1g); these patches have variable shapes, as measured in cat visual cortex^10^. However, in the non-critical regime, the STA-${V}_{{\mathrm{m}}}$ patterns are quite regular (Supplementary Fig. 6a, b). The same seed with different initial conditions produces the similar spatial structure of STA-${V}_{{\mathrm{m}}}$ pattern in the critical regime (Supplementary Fig. 6c,d). We measure the distance between neighboring patches in Fig. 2 of ref. ^10^, which is around 0.79 mm. As one grid unit in our model is considered to be $6.1\times 1{0}^{-3}$ mm (see Methods section), the average distance between neighboring patches in our STA-${V}_{{\mathrm{m}}}$ is 0.61 $\pm$ 0.09 mm, comparable to that measured in ref. ^10^.

**Fig. 2:**
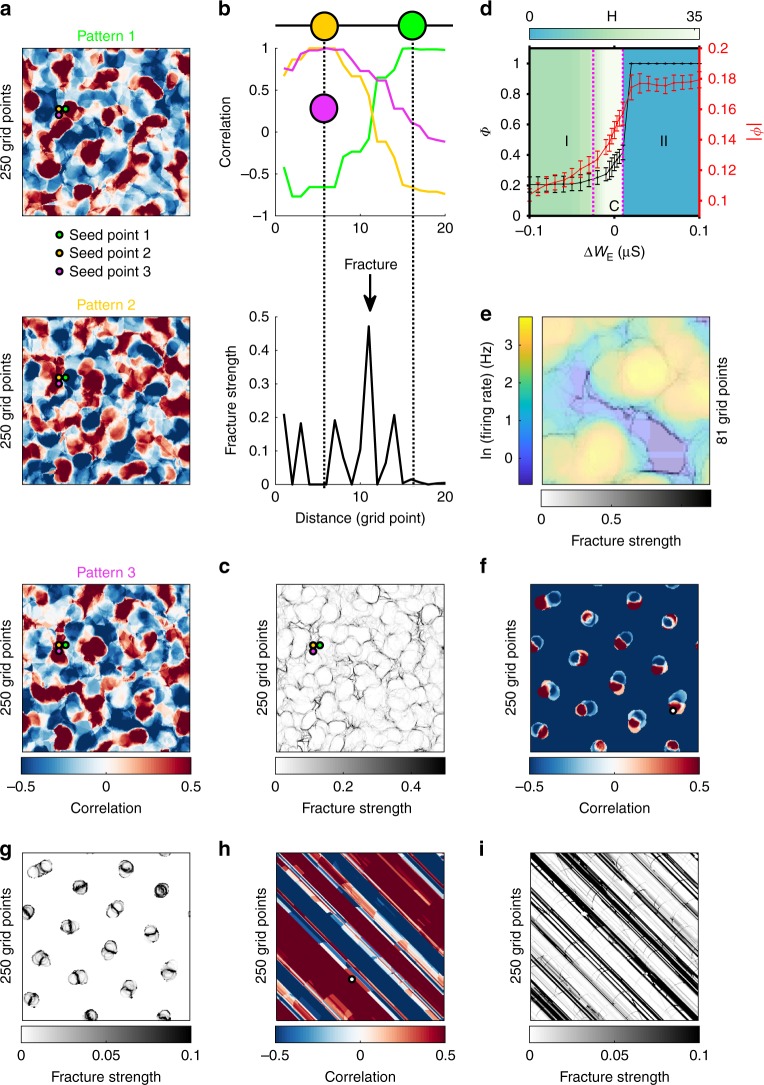
Spontaneous coherent activity with criticality reveals long-range correlation patterns with irregular structures. **a** Spontaneous activity correlation patterns based on two close seed points can be totally different (seed point 1 and 2) or similar (seed point 2 and 3). **b** Moving the seed-point reveals a punctuated rapid transition in global correlation structure expressed by a high rate of change in the correlation pattern between adjacent points (seed point 1 and 2), termed as fracture. Different seeds also can give rise to the similar correlation patterns (seed point 2 and 3). The symbols and colors are the same as in **a**. **c** The fracture generated by systematically moving the seed point when the neural circuit is in the critical regime. **d** The order parameter of collective motion (black) and the pattern shape-based order parameter (red) as the function of $\Delta {W}_{{\rm{E}}}$ (${W}_{{\rm{E}}}={W}_{{\rm{E}}}^{{\rm{original}}}+\Delta {W}_{{\rm{E}}}$). The cyan-white colormap encodes the heterogeneity of dynamical activity patterns. I, C, and II represent randomly wandering patchy pattern, critical, and regular wave states, respectively. The error bars represent s.d. **e** The fracture is consistent with the boundary of high and low firing patches. The semi-transparent fracture map (gray) covers on the firing rate map in 2 s (${\mathrm{ln}}\,({\rm{firing}}\ {\rm{rate}})$ where ${\mathrm{ln}}$ is the natural logarithm.) encoded by the blue-yellow colormap. **f** Shifting the network to the state with regular patchy patterns gives rise to regular correlation patterns. The white circle with black edge labels the seed point. **g** Regular fractures of the state with regular patchy patterns. **h**, **i** Same as in **f**, **g** but for the state with regular crescent waves. Source data are provided as a Source Data file

To further quantify such coherent patterns, we calculate the seed-based correlation map of firing rates (see Methods section)^7^. As shown in Fig. 2a, the correlation map shows a modular organization with patches of positively correlated activity separated by patches of negatively correlated activity; these correlated patchy patterns are notably anisotropic with variable size and shape (Fig. 2a), similar to the spatial structure of correlated spontaneous activity recently observed in ferret visual cortex^7^. To further demonstrate such similarity, as in ref. ^7^, we calculate the rate of change of correlation patterns (see Methods section) as the seed point is moved smoothly across the circuit. Figure 2a shows the correlation patterns when the seed point is moved a small distance across three neighboring points (i.e., Seed point 1, 2, and 3); the correlation patterns for seed point 2 and 3 are very similar to each other, so the rate of change is small ($\sim$0.02, Fig. 2a, b). However, from seed point 1 to 2, the rate change is much greater ($\sim$0.47), as the corresponding correlation patterns based on these two seed points are quite different (Fig. 2a, b). Because of such a sudden change of correlation patterns occurring over a small distance, this phenomenon was referred to as spontaneous fracture^7^. As in ref. ^7^, moving the seed point across the 2D circuit reveals that such spontaneous fractures are organized along some stripes (Fig. 2c). Note that the spatial layout of such fracture stripes is anisotropic, and the lengths of fragmented individual stripes are variable. Similar characteristics of these fracture stripes have been found in ferret visual cortex^7^ (see their Fig. 3).

This anisotropy of such correlation patterns has been explained by heterogeneous local connections in firing rate network models^7^. However, in our model with homogeneous connections (see Methods section), heterogeneous pattern dynamics give rise to this anisotropy. To illustrate this, we introduce an order parameter $\Phi$ based on the collective motion of propagating patterns (Eq. 7, see Methods section). Figure 2d shows that as $\Delta {W}_{{\mathrm{E}}}$ (see Methods section) increases, ${\it{\Phi}}$ changes from small values (~0.21) quantifying the random motion of the patchy patterns to the value of 1, indicating the directed regular propagation of the crescent waves; in the critical regime, $\Phi$ changes rapidly as $\Delta {W}_{{\mathrm{E}}}$ varies. Ramping up and down the parameter $\Delta {W}_{{\mathrm{E}}}$ with different ensembles of initial conditions generates the same curve of $\Phi$ versus $\Delta {W}_{{\mathrm{E}}}$, thus ruling out the possibility of the presence of bistability in our model. As $\Delta {W}_{{\mathrm{E}}}$ increases, the order parameter $| \phi |$ based on the circular symmetry of the pattern shape (Eq. 6, see Methods section) increases and it changes rapidly in the critical regime. This shows that the shapes of the local activity patterns change from a circular to a crescent shape, indicating the existence of symmetry breaking. Such symmetry breaking produces the heterogeneous propagating dynamics in the critical regime, namely that patchy patterns slowly wander around and crescent-shape waves move rapidly (Fig. 1a, Supplementary Movie 1). This situation of heterogeneous dynamics emerging from homogeneous systems due to symmetry breaking is analogous to symmetry breaking-induced heterogeneous dynamics in other homogeneous physical systems^33^. To quantify such heterogeneous dynamics of the activity patterns, we use the index $H$ based on the variability of their propagating speed (Eq. 8, see Methods section) and find that it is maximal in the critical regime (Fig. 2d), indicating the presence of the greatest heterogeneous dynamics.

We next illustrate that the heterogeneous propagating dynamics give rise to the irregular shapes of the correlation map and its fracture structure. Due to the slow movement property of the patchy patterns, the neurons in the areas covered by these patterns have higher firing rates than those outside. However, because the crescent-shaped wave pattern propagates rapidly, it leaves each location quickly and causes only a fraction of neurons in the location to fire, thus generating relatively lower firing rates. In addition, we find that neurons with high firing rates in the areas covered by the patchy patterns are more correlated with each other than neurons outside, suggesting that the boundaries of the patchy patterns tend to have higher fracture strengths; as shown in Fig. 2e, this is indeed the case. Because of the irregular spatial organization of the patchy patterns intermingling with the crescent-shaped waves (Fig. 1a), the fractural strips that have high fracture strengths are spatially anisotropic, as shown in Fig. 2c,e. However, if the network state is shifted away from the critical regime, both the correlation patterns and the shape of fracture exhibit unrealistic, regular spatial structures (Fig. 2f-i), not consistent with experimental observations.

In summary, we have related propagating wave patterns to criticality and demonstrated that such critical activity patterns are the dynamical mechanism underlying the emergent, modular correlation patterns which exhibit spatial heterogeneity.

### Modulation processes of spontaneous activity by stimuli

We next characterize how these spontaneous propagating patterns with critical dynamics are modulated by external stimuli to give rise to stimulus-related neural responses, and reveal the dynamical nature of such modulation processes. To this end, we use natural stimuli such as faces (see Methods section) because they contain rich features^34^ which are necessary for elucidating the functional importance of co-activated activity patterns and for relating them to the classical psychophysical observations of perceptual bubbles in face recognition tasks^15^.

**Fig. 3:**
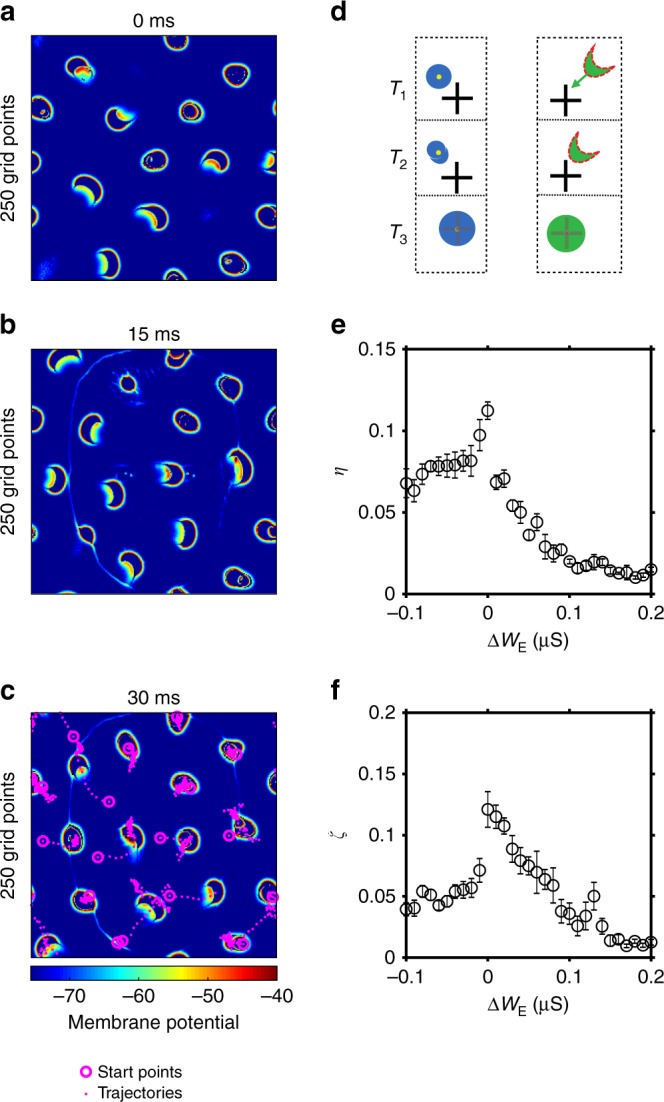
After the onset of stimuli, stimuli modulate spontaneous patterns and the modulation is maximized in the critical point. **a**–**c** Snapshots of membrane potentials after stimulus onset show that most spontaneous patterns do not disappear but their shape and positions are modulated. The dashed lines and circles in **c** are trajectories and start points of the patterns, respectively, since the stimulus onset. **d** Schematic diagram of the modulating process. The cross symbol represents RoIs. Circles with yellow dots signify the patchy patterns. Crescent with red dashed lines represents crescent-shaped waves. ${T}_{1}$ is the moment just before the onset of the stimulus, and ${T}_{2,3}$ are the following time moments. The left panel shows the scenarios of patchy patterns. If the patchy pattern is close to an RoI, the patchy pattern is gradually dragged to the RoI, otherwise not. The right panel shows the scenario of a crescent-shaped wave. If the wave moves toward an RoI, it can be modulated. **e** The modulation index based on the order parameter ($\eta$, Eq. 17) varies as the excitatory coupling strength changes, i.e., ${W}_{{\rm{E}}}={W}_{{\rm{E}}}^{{\rm{original}}}+\Delta {W}_{{\rm{E}}}$. It has a maximum value at the critical point. **f** The modulation index of firing rates ($\zeta$, Eq. 16) versus the excitatory coupling changes. $\zeta$ also has a maximum value at the critical point. The error bars in **e** and **f** represent s.e.m. (standard error of mean). Source data are provided as a Source Data file

Figure 3a–c show that after stimulus onset, most spontaneous patterns do not disappear; instead, they interact with external stimuli such that their shapes and propagating paths are modulated. Specifically, a patchy pattern is gradually shifted or redistributed to an area with higher external current ${I}_{{\mathrm{i}},{\mathrm{j}}}$ (Eq. 1) such as the eyes, denoted as regions of interests (RoIs). During this process, the pattern remains spatially localized, but its shape is changed from a bubble-like to a crescent shape, indicating the existence of symmetry breaking. Subsequently, it is trapped in the RoI, and its shape is restored to a circle (Fig. 3d left), indicating a symmetry restoration process. This modulation process is further illustrated in the snapshots of activity patterns at different time moments after stimulus onset (Supplementary Fig. 7a and Supplementary Movie 1). Figure 3d (right) and Supplementary Fig. 7b show a similar modulation process for a crescent-shaped wave; if it is close or moves toward an RoI, it is shifted to the RoI and then trapped around this RoI, with its shape changed to a circle as well. These patterns modulated or redistributed by external inputs thus underlie stimulus-related responses; we term this dynamical mechanism of modulating spontaneous activity patterns for producing neural responses as Computing by Modulating Spontaneous Activity (CMSA). We find that in our model, 80.05 $\pm$ 14.56% (mean$\pm$s.d.) of stimulus-related responses are due to the modulation process; this is largely consistent with the empirical observation that in the primary visual cortex of cats and mice, 60–80% of the neural responses to stimuli are due to spontaneous activity other than feedforward inputs arising from LGN^1,34,35^. Note that the CMSA mechanism of stimulus-related responses is not restricted to our particular choice of spiking neural circuit models and natural stimuli; CMSA in firing rate models with propagating activity patterns is similarly responsible for stimulus-related responses (see Supplementary Note 1, Supplementary Figs. 16 and 17).

The critical dynamics of the spontaneous coherent activity patterns are essential for implementing the CMSA mechanism. To illustrate this, we introduce a modulation index $\eta$ based on the local order parameter (Eq. 16, see Methods section), by considering the changes of the patterns’ shapes during the modulation process (Fig. 3a–d). If the excitatory coupling strength is increased to shift the state of the circuit away from criticality, our circuit model yields multiple patchy patterns whose positions and shapes cannot be modulated by the input if they are far from the RoIs (i.e., they can only be modulated if they are close to the RoIs), so the order parameter-based modulation index is smaller than that of the critical state. With decreased excitation, the regularly moving waves are not modulated by the stimuli. Indeed, by systematically varying the default excitatory coupling strength, we find that the modulation index is maximal in the critical transition point (Fig. 3e). Furthermore, the firing-rate-based modulation index ($\xi$, see Methods section) also demonstrates that the modulation effect is maximal in the critical transition regime (Fig. 3f).

We now demonstrate that the CMSA-based neural responses have smaller variability than spontaneous activity. We first calculate the mean-matched Fano Factor of spike count (see Methods section) after stimulus onset for each neuron in the local area around RoIs, and find that the decline of Fano Factor has an M-shaped structure (Supplementary Fig. 8a), as found in the middle temporal (MT) area of monkeys^36^. We then calculate the average Fano Factor of all neurons after the stimulus onset; as shown in Supplementary Fig. 8b, there is a decline in the Fano Factor values, as observed in experiments^30^. This quenching of neural variability in our model is mainly due to the modulated pattern dynamics after stimulus onset. During spontaneous activity, the movements of the slow patchy patterns and the fast wave patterns lead to dynamical transitions between low and high activity states and the resultant firing rate fluctuations. However, once the patterns are modulated to the RoIs, they are trapped (Fig. 3c, Supplementary Fig. 7), thus neurons in the RoIs fire with higher rates without the fluctuating transitions to the low activity state as during the spontaneous activity, consequently resulting in the decline of neural variability.

**Fig. 4:**
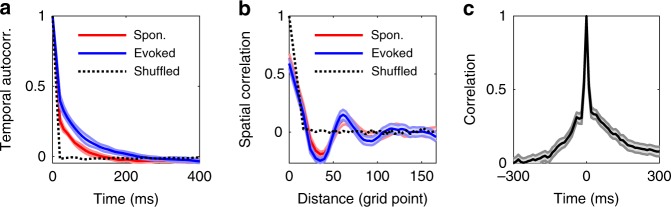
Stimuli slightly modulate the temporal autocorrelation and spatial correlation. **a** The averaged temporal autocorrelation of instantaneous firing rates (bin width: 20 ms, randomly selected neurons) of spontaneous and evoked activities. The temporal correlation of evoked activity decays more slowly than that of spontaneous activity. The black dashed lines in **a, b** are correlations for shuffled responses. The lighter shaded lines represent ten s.e.m. Lines are an average over an ensemble of ten trials with random initial conditions at each point. **b** Average correlation coefficients of the instantaneous firing rate (bin width: 50 ms), computed over all neuron pairs in RoIs, as a function of their distance. The lighter shaded lines represent s.e.m. Lines are an average over an ensemble of ten trials with random initial conditions at each point. **c** The 2D Pearsons correlation between the smoothed ${V}_{{\rm{m}}}$ frame just before the stimulus onset and other frames every 0.1 ms from −300 to 300 ms (0 ms is the moment just before stimulus onset). The lighter shaded lines represent 3 s.e.m. Lines are an average over an ensemble of 180 trials with random initial conditions at each point. Spon.: Spontaneous activity, Evoked: Evoked activity, Shuffled: shuffled activity, i.e., randomly shuffling every neuron’s activity; Autocorr., Autocorrelation. Source data are provided as a Source Data file

In addition, our CMSA mechanism can account for slight modulations of temporal and spatial correlations of neural activity. As shown in Fig. 4a, b, both spatial and temporal correlations of evoked activity have similar spatial structures to the spontaneous activity and are comparable to those found in the visual cortex of awake ferrets^2^. We further calculate 2D Pearson correlations between the frames of membrane potential before and after stimulus onset^1^; the decay time constant ($\sim$20 ms) of the two halves of the correlation curves as shown in Fig. 4c are similar, consistent with that of $\sim$18 ms found in the visual cortex of awake ferrets^2^. These results thus indicate that external inputs only slightly modulate spontaneous neural correlations.

### The CMSA mechanism speeds up cortical processing

**Fig. 5:**
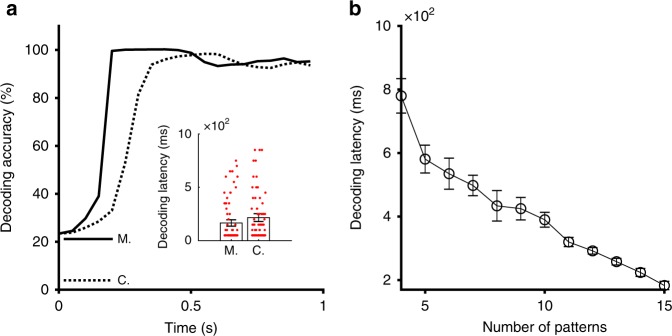
Spontaneous activity patterns speed up the stimulus-related response. **a** In a typical example, decoding analyses show that the response of the CMSA case (M.) is faster to attain significant decoding accuracy than that of control case (C., the preceding spontaneous activities are randomly shuffled). Inset: the statistic of two conditions’ decoding latency. Red dots are data points. 0 s is just before the stimulus onset. The error bars represent 3 s.e.m. **b** The co-activation property of the activity patterns benefit the modulation processes. The decoding latency of modulation processes decreases with the increase of the number of patterns in the network. The error bars represent s.e.m.; data is averaged over an ensemble of ten trials with random initial conditions at each point. Source data are provided as a Source Data file

We next illustrate the consequence of the CMSA mechanism for neural processing by decoding analysis (see population decoding analysis in see Methods section)^37^. A classifier is used to assess the information about the stimuli encoded in the neural activity under two conditions: the CMSA and the control cases by randomly shuffling membrane potentials (see Methods section). The decoding analysis demonstrates that face-stimulus identities are encoded well in both conditions, reaching high accuracy ($> 90 \%$) after a few hundred milliseconds (Fig. 5a). However, we find the decoding accuracy increases significantly faster in the CMSA case than in the control case ($p=1.5\times 1{0}^{-3}$, lower-tail *t*-test, Fig. 5a inset); their mean decoding latencies are 165.9 ms and 216.4 ms across 250 independent trials, respectively. This result thus indicates that the CMSA mechanism can speed up processing external inputs. It is interesting to note that co-activation property of spontaneous patterns means that they can be modulated simultaneously by the stimuli, as shown in Fig. 3a–c, indicating that the modulation process occurs in a fundamentally distributed and parallel way. The parallel modulation thus would contribute to the acceleration effect of the CMSA mechanism. To test this, we randomly reshuffle the membrane potentials over neurons in real-time in some selected regions of the network to decrease the number of spontaneous coherent activity patterns in the network, and implement the decoding analysis. As shown in Fig. 5b, for fewer patterns, the decoding latency increases. More patterns cause more interactions of co-activated patterns such as collisions which provide more opportunities for spontaneous patterns to be trapped by stimuli.

To further demonstrate the speeding-up effect of the CMSA mechanism, we calculate the decoding latency by systematically varying the default excitatory coupling strength; as shown in Supplementary Fig. 9a, the latency is shortest in the critical regime. In addition, we calculate the decoding accuracy at 100 ms after the stimulus onset with varying excitatory coupling strength, and find that the accuracy is the highest in the critical regime (Supplementary Fig. 9b).

### The CMSA underlies the variability in neural responses

Intracellular recordings have shown that neural response variability is attributable largely to coherent fluctuations in spontaneous activity in cat striate cortex^5^. Specifically, the trial-by-trial fluctuations of spontaneous membrane potential (${V}_{{\mathrm{m}}}$) are significantly correlated to those of the rate and latency of action potentials during epochs after the stimulus onset^5^. We now demonstrate that this fundamental relationship between spontaneous activity and evoked neural responses can be reproduced in the critical regime of our model, and the CMSA mechanism provides an explanation for this relationship.

**Fig. 6:**
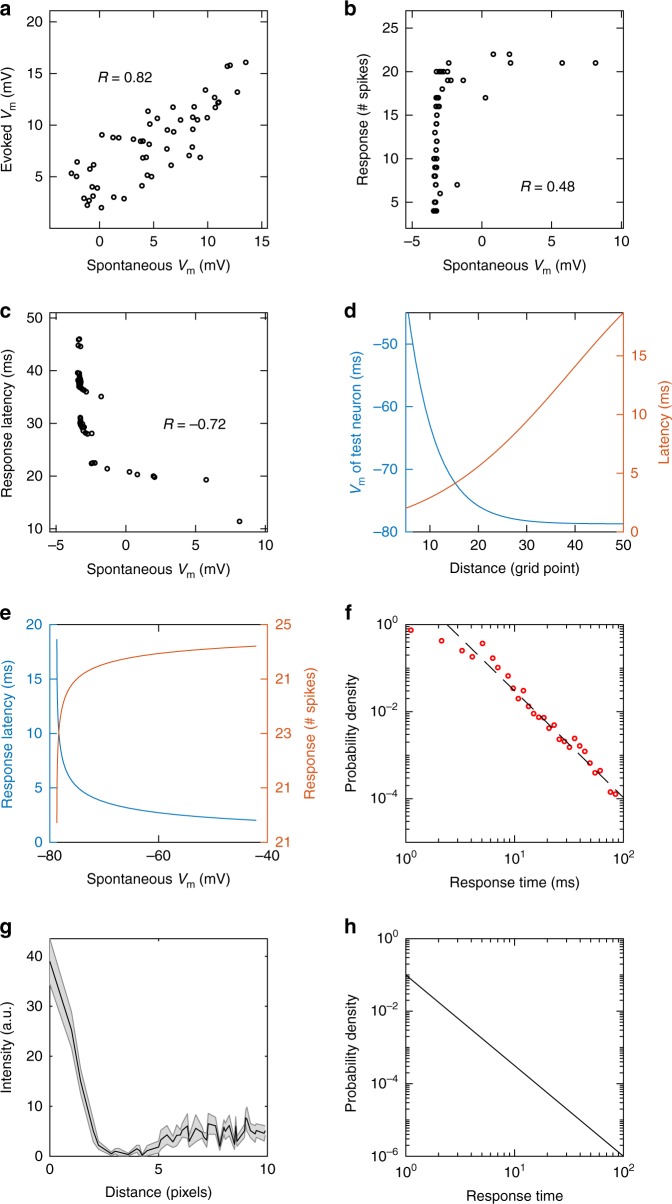
CMSA explains the relations between spontaneous and evoked activities and response variability. **a** Scatter plot of the mean ${V}_{{\rm{m}}}$ of the evoked response over a period of $T$ (2–120 ms, mean value: 8 ms) after the stimulus onset versus its preceding spontaneous mean ${V}_{{\rm{m}}}$ over $T$ immediately before the stimulus onset. $T$ is the duration of the modulation process, that is, the time interval from the stimulus onset to the time when the spontaneous pattern is trapped. **b** Scatter plot of the number of spikes occurring in the first $2T$ of the response and the mean spontaneous ${V}_{{\rm{m}}}$ in the $T$ preceding the stimulus onset. **c** Scatter plot of latency to the first spike and the spontaneous ${V}_{{\rm{m}}}$. **d** Spontaneous ${V}_{{\rm{m}}}$ and latency of neurons depend on the distance between the ongoing pattern and the test neuron. These curves are smoothed by Gaussian-weighted moving average over each window. **e** Latency and number of spikes of the test neuron versus spontaneous ${V}_{{\rm{m}}}$. The curves are also smoothed as in **d**. **f** The distribution of response times in simulations. The tail of the distribution of response time can be fitted as a power-law function (dashed line, exponent: −2.5). **g** The pixel intensity of face stimuli (filtered by DoG) decays as the distance from the RoIs increases. The shaded area represent 3 s.d.; the line averages over an ensemble of 16 face images. **h** The distribution of response times in the stochastic model. Source data are provided as a Source Data file

We randomly sample neurons in the areas with the modulated patterns and calculate their average ${V}_{{\mathrm{m}}}$ before and after stimulus onset within a time interval of $T$ ms (2–120 ms, mean value: 8 ms), which is the duration of the modulation process, that is, the time interval from the stimulus onset to the time when the spontaneous pattern is trapped (Fig. 3a–c). We sample 52 neurons to match the number of neurons sampled in ref. ^5^, but sampling different numbers of neurons yield similar results. As shown in Fig. 6a, the adjacent epochs of spontaneous and evoked ${V}_{{\mathrm{m}}}$ are significantly correlated ($r=0.59\pm 0.11$, mean $\pm$ s.d.; 76.67% neurons). In addition, on each trial, we calculate the latency of the first spike and the number of spikes occurring in the first $2T$ ms after stimulus onset and compare these values with the mean ${V}_{{\mathrm{m}}}$ occurring during the $T$ ms period preceding stimulus onset. An example of this analysis from a typical trial is shown in Fig. 6b, c; the number of spikes of each sampled neuron and its spiking latency shows considerable variability, ranging from 0 to 37 spikes, and 0.8 ms to 105.8 ms, respectively. The number of spikes increases and the latency of the first spike decreases in proportion to the ${V}_{{\mathrm{m}}}$ preceding stimulus onset (Fig. 6b, c), with the mean values of the correlation coefficients of $0.55\pm 0.11$ and $-0.49\pm 0.12$, respectively. These correlation values are quantitatively comparable to those reported in cat visual cortex^5^, namely, the values of $0.56\pm 0.15$ and $-0.61\pm 0.18$ for the spiking number and latency to ${V}_{{\mathrm{m}}}$ correlations, respectively. Also, by shuffling spontaneous ${V}_{{\mathrm{m}}}$ among neurons, we find that the shuffled activity is not correlated to the evoked ${V}_{{\mathrm{m}}}$, spike number and spike latency (correlation: $0.01\pm 0.02$, Supplementary Fig. 10), indicating the variability of neural responses is indeed correlated to the underlying fluctuations of spontaneous activity. However, if the network is shifted away from the critical transition (Supplementary Fig. 1b, c), there are lower correlations between spontaneous ${V}_{{\mathrm{m}}}$ and neural responses evoked by stimuli (Supplementary Fig. 11), suggesting that the propagating patterns with criticality are essential not only for accounting for the key spatiotemporal properties of coherent activity but also for understanding their functional mechanism.

We next illustrate that the modulation process of spontaneous activity patterns provides a mechanistic explanation for the relationship between the spontaneous ${V}_{{\mathrm{m}}}$ and evoked activity. It is apparent that a localized moving pattern, which includes multiple spiking neurons, provides a source of synaptic inputs to neurons in the area that it approaches, resulting in depolarization of their membrane potentials due to the recurrent synaptic interactions (see Methods section). Because such synaptic interactions are distance-dependent, the magnitudes of depolarization depend on the distance between the activity pattern and the neurons; namely, shorter distances lead to greater depolarization of the spontaneous ${V}_{{\mathrm{m}}}$ activity. The time interval for the pattern to reach the trapped point should be positively related to the distance as well. To verify this, we track the spontaneous activity patterns until they are trapped by the modulation process and calculate how these spontaneous patterns are related to the depolarization magnitude of ${V}_{{\mathrm{m}}}$ of a test neuron. Figure 6d shows that indeed, the spontaneous ${V}_{{\mathrm{m}}}$ of the test neuron increases as the pattern moves closer to it, resulting in shorter spike latency. Arranging the latency and spike count of the test neuron with respect to spontaneous ${V}_{{\mathrm{m}}}$ (Fig. 6e), we find the relationships between these spiking properties and ${V}_{{\mathrm{m}}}$ are similar statistical results (Fig. 6b, c). Therefore, the pattern dynamics in the modulation process contribute to the significant correlations between spontaneous activity and evoked responses, as shown in Fig. 6a–c. The noisy features of the scattered points in Fig. 6a–c arise from differences in the number of spikes within different moving patterns and the complex trajectories of these patterns, which often do not approach a neuron head-on but move within its proximity. In addition, approximately 23% of neurons do not have such significant correlations between spontaneous and evoked activity; it is interesting to note that in ref. ^5^, a similar proportion of sampled neurons did not show such correlations. In our model, this happens mainly because $\sim$80% but not all of the stimulus-related responses arise from the modulation process in our model.

To further quantify the trial-to-trial variability of evoked responses, we calculate the response time for an ongoing activity pattern to be modulated to the RoIs after the stimulus onset. The response time has great variability, as evidenced by the power-law tail of its distribution (Fig. 6f). Such variable neural responses occur because different trials have different configurations of spontaneous correlated patterns and these patterns propagate in a seemingly random way with different trajectories and distances to the RoIs.

### Analysis of the CMSA process

To gain further theoretical insights into how the CMSA mechanism underlies variable neural responses, we develop a simple yet effective stochastic model that captures the key dynamical properties of the modulation process. Due to the random, complex motion of each pattern, we approximate it as a random walker in the stochastic model. The pixel intensity values of DoG-filtered face images (see Methods section) near the RoIs (such as eyes) decay as the distance to the RoIs center increases (Fig. 6g). Such a structure of inputs would result in the case that the closer the neuron is to the center, the more it is depolarized. If a wave pattern propagates into such a depolarization gradient, the amplitude of excitatory inputs to the pattern would be gradually increased (Eq. 1); this means that the walker in the stochastic model receives a pulling force shifting it to the center of the region, and this force increases as the pattern moves towards the center. To model a random walker with such a force, we use the stochastic model of a random walker moving in a logarithmic potential^38^ (see Supplementary Note 2).

By analytically solving this stochastic model, we find that the time interval for the walker to be shifted to the center of the potential well is very variable; the interval depends on the distance between the initial position and the center of the well, and the distribution is a power-law function (Fig. 6h), consistent with our results of the spiking neural circuit (Fig. 6f). Here it is interesting to note that such a power-law distribution of reaction time has been found in perceptual and cognitive functions^39^; this consistency suggests that the power-law neural response time from CMSA might be the neurophysiological mechanism underlying these behavioral observations^39^. The theoretical model, which captures the key dynamical properties of propagating patterns, further elucidates the modulation process is essential for understanding the variability of neural responses.

### The CMSA mechanism explains perceptual psychophysics

In psychophysics studies, it has been found that a small number of localized patches of a complex visual stimulus such as a face would reveal enough information for recognizing the face; these patterns are thus referred to as perceptual bubbles^15^. The perceptual bubbles found in psychophysical experiments of face recognition tasks cover eyes, nose and mouth (Supplementary Fig. 12). We now show that the face-evoked neural response patterns due to the CMSA mechanism are highly correlated to perceptual bubbles, thus suggesting a neurophysiological mechanism underlying the psychophysical observation of perceptual bubbles.

**Fig. 7:**
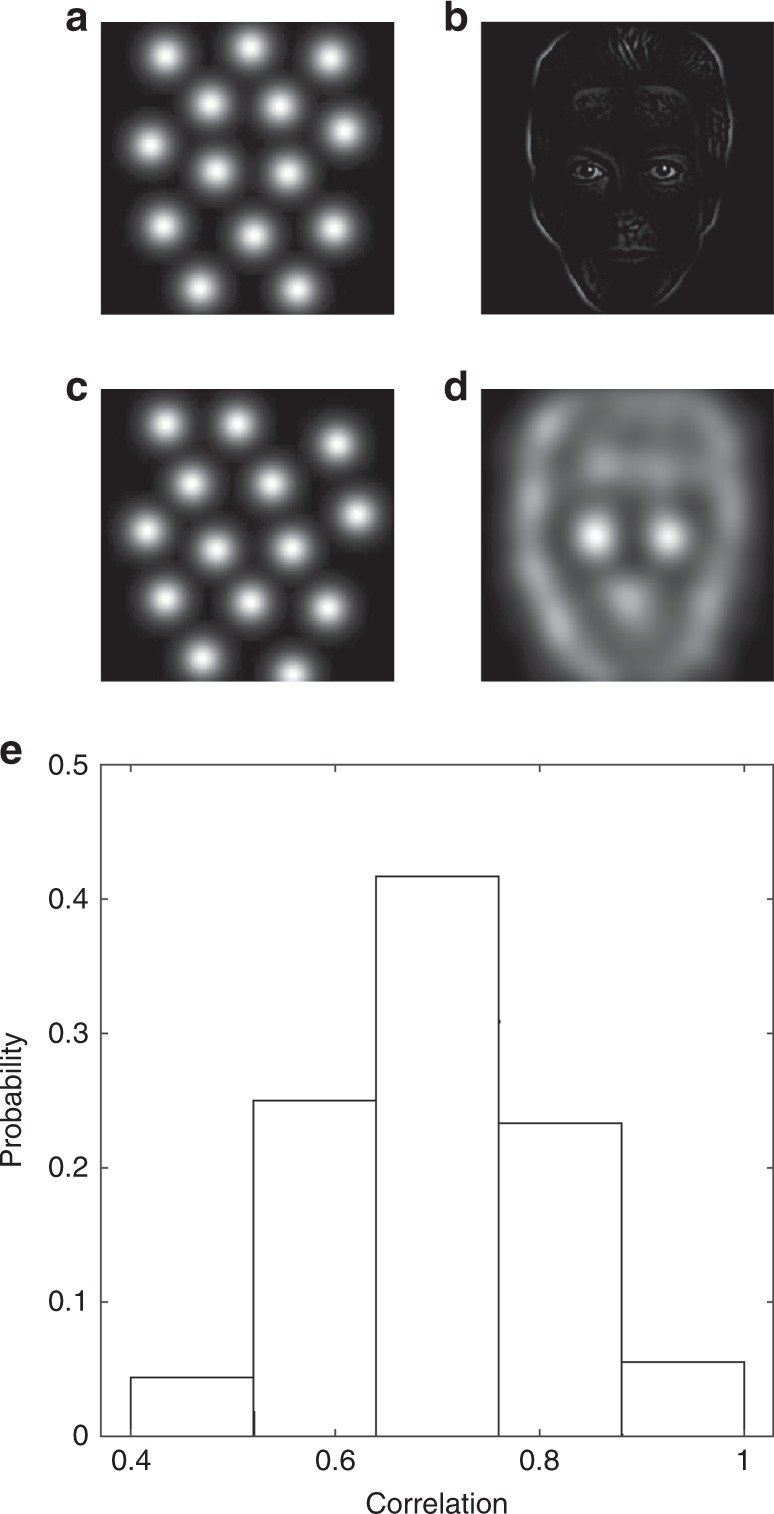
Similarity of the bubbles selected by the ideal observer and the modulated activity patterns. **a** The best bubble mask selected by the ideal observer. **b** The diagnostic face generated by the bubble mask in **a** exposes the eyes, mouth, and outline. **c** The 2D converted map of modulated patterns in a single trial is similar to the best bubble mask. **d** The averaged 2D converted map of modulated patterns over 100 trials is similar to the best bubble mask. **e** The histogram of trial-by-trial correlation between the bubbles selected by the ideal observer and 2D converted maps among 100 trials (mean value: 0.67). Source data are provided as a Source Data file

As in ref. ^15^, to generate a configuration of bubble representations of a face, we use an ideal observer that is exposed to the face punctuated with bubble masks. The bubbles generated by the ideal observer provide a benchmark of the information available in the stimulus set (i.e., faces) for the task at hand, and is similar to those used by human observations (see Methods section). The bubbles selected by the ideal observer are shown in Fig. 7a; they expose the regions that have the highest local variance over all faces, which are the most distinguishable parts for face identities and cover the eyes, nose, mouth, and the outline of a face (Fig. 7b). In our circuit model, the regions covered by trapped localized patterns after the modulation processes (Fig. 3c) are similar to these perceptual bubbles.

To directly compare the trapped patterns in our spiking model and the perceptual bubbles selected by the ideal observer, we use the similar number of trapped patterns and bubbles, and convert each trapped pattern to a 2D Gaussian shape (Fig. 7c, see Methods section). We then calculate the 2D correlation between the average converted map of evoked patterns across trials (Fig. 7d) and the 2D perceptual bubble pattern selected by the ideal observer (Fig. 7a), and find that they are highly correlated (0.65); their trial-by-trial correlations are variable but have a large mean value (0.67 $\pm$ 0.11, mean $\pm$ s.d., Fig. 7e). Furthermore, we find such correlations due to the CMSA mechanism are maximal in the critical regime (Supplementary Fig. 13).

### The effect of noise on the CMSA mechanism

We have mainly focused on studying how the CMSA intrinsically emerges in the circuit models with constant inputs. However, the key properties of CMSA, such as the critical pattern dynamics and the modulation process in response to inputs are robust to external noisy perturbations. To illustrate this, we add different types of noise including white, pink noises and random Poisson spikes to our model (see Methods section) and then do the same analyses as done above for the deterministic model. We find that for relatively small noise intensity values ($\sigma\,<\,0.02\ {\mathrm{\mu}}{\mathrm{S}}$) for white noise, although the activity patterns appear to be noisier, their critical dynamics can persist (Supplementary Fig. 14a–c); as does the modulation index (Supplementary Fig. 14d). Thus the characteristic features of the CMSA mechanism are robust to modest randomness of external perturbations. From the noise intensity values of 0.02 to 0.06, there is a rapid transition to the regime of localized, patchy patterns; beyond noise intensity 0.06, there are only patchy patterns without long-range propagation (Supplementary Fig. 14e, f). The modulation effect thus recedes as the noise intensity increases. These results suggest that noise perturbations tend to make the moving dynamics more spatially constrained; we observe a similar trend for the model with pink noise. We also added Poisson spikes on every neuron with sparse firing rate $f$, and find that the CMSA persist for $f\,<\,2\,{\mathrm{Hz}}$ (Supplementary Fig. 14g, h).

## Discussion

In this study, we have established a novel neural computational mechanism (CMSA) to understand how cortical circuits process sensory inputs. The CMSA mechanism exploits rich spatiotemporal dynamics of spontaneous activity to actively speed up the processing of external inputs, rather than directly responding to inputs as in conventional neural computation models such as attractor neural networks^40,41^ and Gabor filter-based models widely used for understanding sensory responses^34,42^ and for predicting psychophysical performance^42,43^. As we have demonstrated, the CMSA mechanism provides a unified account of a wide range of key properties of spontaneous coherent activity patterns^2,7,10^, input-related responses^2,5^ and their fundamental relations^2^ found in experimental studies; these properties would otherwise remain unexplained in existing models of cortical processing^32,41,44,45^. The CMSA mechanism makes new predictions of cortical processing and suggests that it might be a general computational property of cortical circuits.

In our CMSA model, the effect of modulation from external inputs on ongoing coherent patterns is maximal when these patterns possess critical dynamics. As we have demonstrated, spontaneous coherent patterns with criticality capture two key properties of ongoing activity patterns, i.e., their co-activated^10^ and propagation properties^12^. Co-activated, coherent activity patterns, as detected by the spike-triggered average analysis^1^ and a seed-based cross-correlation analysis^8,9,11^, have been widely observed at both the neural circuit and the whole-brain levels; our findings are consistent with these previous reports, but go beyond them by revealing the functional impact of the co-activation property. That is, these co-activated patterns can be modulated by natural inputs in response to their multiple salient features in a fundamentally distributed and parallel manner. Such a distributed functional mechanism of co-activated patterns is better illustrated by using natural stimuli, thus supporting the argument that the use of natural stimuli is vital for uncovering the computational principles of the cortex^34^.

These coherent patterns have the propagation property, a cortical activity mode that has been widely observed across a diverse range of cortical areas^12,13^. As we have demonstrated, these propagating patterns exhibit critical dynamics characterized by their size and duration distributions, the maximal dynamic range, and the correlation length of pattern velocity fluctuations that scales linearly with the size of the network. Our results thus relate wave patterns with criticality, beyond previous studies that have mainly focused on stable waves^46,47^ (but see ref. ^48^ for a study of metastable waves). This unique angle of studying neural criticality is analogous to decomposing avalanches into propagating waves to reveal the spatiotemporal nature of self-organizing criticality in complex physical systems^49^. Recent studies have been increasingly proposing that criticality of cortical dynamics could not be a quiescent-to-active phase transition as usually assumed in theoretical approaches^50^, but rather being close to the transition between different cortical states (i.e., asynchronous and synchronous/coherent states)^51–55^. Particularly, it is interesting to note that in these studies, the existence of propagating activity patterns in their circuit models with spatially extended connectivity like ours has been clearly demonstrated in a critical regime (see Fig. 2 of refs. ^52–54,55^). Given that spatially extended, distance-dependence coupling has been widely observed over the multiple scales of neural systems^20,56^, it would be important to further study spatiotemporal organization of critical brain dynamics from the perspective of propagating activity patterns using the methods introduced in this study, such as the method based on correlation lengths of pattern velocity fluctuations.

Propagating patterns with heterogeneous dynamics (i.e., localized activity patterns with different shapes and different propagation speeds) emerge in the critical transition regime of our circuit model due to symmetry breaking as characterized by the order parameters. As we have illustrated, heterogeneous pattern dynamics can account for patchy coherent structures with heterogeneous spatial profiles, as found in ref. ^7^. These patterns thus provide a dynamical mechanism, different from the heterogeneous connectivity mechanism proposed in ref. ^7^, for explaining how spatially heterogeneous correlated activity can emerge even from homogeneous neural circuits. However, connectivity heterogeneity as used in ref. ^7^ may stretch the critical region of these correlated patterns by the mechanism of the Griffiths phase^57^, thus giving rise to such patterns in a fundamentally robust way.

In order to test propagating patterns with criticality as predicted in our study, it would be relevant to use the same methods as in our modeling study to analyze their statistical scaling property in large scale recording with high spatial and temporal resolutions. Spatiotemporally contiguous clusters of active pixels in awake brains of mice have been found to have the same power-law distributions^22^ as in our modeling study; it would be interesting to test whether the spatially and temporally contiguous features of these clusters are due to the propagation of wave patterns across the cortex as predicted in our modeling study. Furthermore, through detailed comparisons between neurophysiological data and our model, we have demonstrated that the CMSA modulation effect is maximal when the spontaneous activity patterns possess criticality and that critical pattern dynamics are essential for active cortical processing. Our study thus contributes to a growing line of research on the functional importance of critical neural dynamics^17,18^ from a novel perspective.

Our CMSA model uncovers the mechanistic relations between neural responses during spontaneous activity and sensory stimulation. In our model, spontaneous propagating patterns move in a seemingly random way and would then be modulated by external sensory inputs; such a modulation process does not destroy these patterns, but re-shapes and re-distributes their moving paths and positions. Therefore, most ($\sim$80%) of the input-related spikes are due to this dynamical process rather than being directly evoked by external inputs, consistent with neurophysiological observations^5^. As we have demonstrated, co-activated, patchy patterns modulated by faces have the bubble-like shapes with the similar spatial structure as the perceptual bubbles used in face recognition tasks^15^. The CMSA mechanism thus relates neural activity patterns to behavioral observations, indicating that neurophysiological and psychophysical attributes of brain function can be understood from a single theoretical framework.

By developing and analyzing the stochastic model that captures the key features of spontaneous activity patterns, we have further illustrated the dynamical nature of the CMSA. Particularly, our theoretical analysis along with simulations of the full spiking neural circuits shows how and why the latency and rates of evoked spikes are related to spontaneous membrane potentials, as observed in intracellular recordings^5^. Aside from accounting for these empirical observations, our CMSA model predicts that fluctuations of neural response latency across trials should be scale-invariant, as evidenced by its power-law distribution. It is interesting to note that such a scale invariance of reaction time fluctuations has been found in a variety of perceptual and cognitive tasks^39^; this consistency leads us to predict that the scale invariance of neural responses originated from modulating the spontaneous activity patterns with criticality might be the neurophysiological mechanism underlying the behavioral scale-invariant fluctuations^39^

This CMSA mechanism has been mainly demonstrated in our study by bottom-up inputs, but the same dynamical mechanism may underlie the effect of top-down inputs as well. Indeed, it has been found that top-down inputs such as expectation and attention modulate spontaneous ongoing activity^37,58^; it thus is of great relevance to ask whether such modulation processes belong to the same active CMSA mechanism as we have revealed in the present study. As has been proposed in previous studies, spontaneous activity contains prior information of natural environments^59^. In light of this proposal, the complex moving dynamics of spontaneous activity patterns may implement an efficient sampling of such prior information, similar to random diffusion-based sampling in the classical Markov Chain Monte Carlo methods^60^. Our CMSA neural computation mechanism, therefore, has the potential to merge probabilistic and dynamical views of cortical processing, providing a framework for understanding how the cortex integrates endogenous priors, top-down attention and sensory inputs to actively produce perception.

## Methods

### A spatially-extended spiking neural circuit

We consider a 2D network of $N\times N$ coupled, conductance-based exponential integrate-and-fire neurons consisting of 75% excitatory and 25% inhibitory neurons; a similar model has been used to model variable neural dynamics^61^ and the change of cortical states due to external stimuli^62^. Both excitatory and inhibitory neurons are evenly spaced, and the spacing between inhibitory neurons is twice the spacing between excitatory neurons. We denote the membrane potential of a neuron at integer coordinates $({\mathrm{i}},{\mathrm{j}})$ at time $t$ as ${V}_{{\mathrm{i}},{\mathrm{j}}}(t)$, with its dynamics given by the following:

1 $$C\frac{{\mathrm{d}}}{{\mathrm{d}}t}{V}_{{\mathrm{i}},{\mathrm{j}}}(t)	= -{g}_{{\mathrm{L}}}[{V}_{{\mathrm{i}},{\mathrm{j}}}(t)-{V}_{{\mathrm{L}}}]+{g}_{{\mathrm{L}}}{\Delta }_{{\mathrm{T}}}\exp \left(\frac{{V}_{{\mathrm{i}},{\mathrm{j}}}(t)-{V}_{{\mathrm{T}}}}{{\Delta }_{{\mathrm{T}}}}\right)\\ 	\hskip 10pt -\,{g}_{{\mathrm{i}},{\mathrm{j}}}^{{\mathrm{E}}}(t)[{V}_{{\mathrm{i}},{\mathrm{j}}}(t)-{V}_{{\mathrm{E}}}]-{g}_{{\mathrm{i}},{\mathrm{j}}}^{{\mathrm{I}}}(t)[{V}_{{\mathrm{i}},{\mathrm{j}}}(t)-{V}_{{\mathrm{I}}}]+{I}_{{\mathrm{i}},{\mathrm{j}}},$$

where the capacitance is $C=1$ nF, the leaky conductance is ${g}_{{\mathrm{L}}}\,=\,0.05\ {\mathrm{\mu}}{\mathrm{S}}$, and the exponential nonlinearity parameters are ${\Delta }_{{\mathrm{T}}}=6.5625$ mV and ${V}_{{\mathrm{T}}}=-60.6250$ mV^63^. The reversal potentials are ${V}_{{\mathrm{L}}}=-70$ mV, ${V}_{{\mathrm{E}}}=0$ mV, and ${V}_{{\mathrm{I}}}=-80$ mV for the leak, excitatory, and inhibitory conductances, respectively^64^. ${I}_{{\mathrm{i}},{\mathrm{j}}}$ is the external input; when ${I}_{{\mathrm{i}},{\mathrm{j}}}=0$ nA, the network exhibits spontaneous activity. The external inputs such as face images are specified below. If the membrane potential of a neuron reaches the threshold, −40 mV, a spike is generated and the voltage is reset to the resting potential −70 mV for a refractory period ${\tau }_{{\mathrm{ref}}}=5$ ms. The synaptic conductances are as follows:

2 $${g}_{{\mathrm{i}},{\mathrm{j}}}^{\lambda }(t)={F}^{\lambda }+\sum _{({\mathrm{i}}^{\prime} {\mathrm{j}}^{\prime} )}{K}_{{\mathrm{i}}{\mathrm{j}},{\mathrm{i}}^{\prime} {\mathrm{j}}^{\prime} }^{\lambda }\sum _{l}{G}^{\lambda }(t-{T}_{{\mathrm{i}}^{\prime} {\mathrm{j}}^{\prime} }^{l}),$$

where superscript $\lambda ={\mathrm{E,I}}$ represents excitatory and inhibitory neurons, respectively, and ${T}_{{\mathrm{i}}^{\prime} {\mathrm{j}}^{\prime} }^{l}$ is the time of the $l$-th spike emitted by the afferent neuron located at $({\mathrm{i}}^{\prime} ,{\mathrm{j}}^{\prime} )$. The inputs are ${F}^{{\mathrm{E}}}\,=\,0.01\ {\mathrm{\mu}}{\mathrm{S}}$ and ${F}^{{\mathrm{I}}}\,=\,0.01\ {\mathrm{\mu}}{\mathrm{S}}$. To consider the effects of noise on network dynamics, we add different types of noise including Gaussian white noise, pink noise and random Poisson spikes. For Gaussian noise, we add a noise term to ${F}^{{\mathrm{E}}}$, i.e., ${F}^{{\mathrm{E}}}\,=\,0.01\,+\,\sigma \xi (t)\ {\mathrm{\mu}}{\mathrm{S}}$, where $\xi (t)$ is the noise drawn from the standard normal distribution and $\sigma$ is the intensity. Similarly, we add pink noise on ${F}^{{\mathrm{E}}}$. For Poisson spikes, we add random spikes to every neuron with a firing rate $f$. The time course of the postsynaptic conductance is given by

3 $${G}^{\lambda }(t)=\frac{\exp (t/{\tau }_{d}^{\lambda })-\exp (t/{\tau }_{{\mathrm{r}}}^{\lambda })}{{\tau }_{{\mathrm{d}}}^{\lambda }-{\tau }_{r}^{\lambda }},$$

with rise times ${\tau }_{{\mathrm{r}}}^{{\mathrm{E}}}=0.3$ ms and ${\tau }_{{\mathrm{r}}}^{{\mathrm{I}}}=0.3$ ms, and decay times ${\tau }_{{\mathrm{d}}}^{{\mathrm{E}}}=2$ ms and ${\tau }_{{\mathrm{d}}}^{{\mathrm{I}}}=3$ ms. The coupling strength between any two neurons located at $({\mathrm{i}},{\mathrm{j}})$ and $({\mathrm{i}}^{\prime} ,{\mathrm{j}}^{\prime} )$ is

4 $${K}_{{\mathrm{i}}{\mathrm{j}},{\mathrm{i}}^{\prime} {\mathrm{j}}^{\prime}}^{\lambda }=\left\{\begin{array}{cc}{W}_{{\mathrm{E}}}\cdot \exp \left(-{d}_{{\mathrm{i}}{\mathrm{j}},{\mathrm{i}}^{\prime} {\mathrm{j}}^{\prime}}^{2}/{\sigma }_{{\mathrm{E}}}\right)& \, {\text{if}}\ \lambda ={\mathrm{E}},\\ {W}_{{\mathrm{I}}} & \, {\text{if}}\ \lambda ={\mathrm{I}},\end{array}\right.$$

where ${d}_{{\mathrm{ij}},{\mathrm{i}}^{\prime} {\mathrm{j}}^{\prime} }$ is the Euclidean distance between neurons located at $({\mathrm{i,j}})$ and $({\mathrm{i}}^{\prime} ,{\mathrm{j}}^{\prime} )$ on a square lattice with periodic boundary conditions. Connections in this model are constrained to $| {d}_{{\mathrm{ij}},{\mathrm{i}}^{\prime} {\mathrm{j}}^{\prime} }|\, \leqslant \, {D}^{\lambda }$, ${D}^{{\mathrm{E}}}=45$ grid points, ${D}^{{\mathrm{I}}}=45$ grid points. This coupling range is biologically plausible as within this range; there are around 6300 synaptic connections per neuron, approximately equivalent to the average number of synapses in the visual cortex, 6000 synapses per neuron^65^. As ${D}^{\lambda }$ increases, the number of patterns and firing rate decreases, and the distance between neighboring patterns increases (Supplementary Fig. 15). Empirical evidence has been accumulating to show that neural connectivity decays with distance; for instance, it was found that the coupling strength is an exponential function of distance^56^; and it was reported that the connection probability of neurons is a Gaussian function of distance^20^. Because anatomic evidence suggests that inhibitory connections to pyramidal neurons are nonspecific^66^, in Eq. 4, we use a homogeneous inhibitory coupling strength ${W}_{{\mathrm{I}}}$ with all inhibitory neurons connected within the coupling range. We use inhibitory coupling value ${W}_{{\mathrm{I}}}\,=\,0.0578\ {\mathrm{\mu}}{\mathrm{S}}$ and excitatory coupling value ${W}_{{\mathrm{E}}}\,=\,0.2235\ {\mathrm{\mu}}{\mathrm{S}}$ with a spatial scale ${\sigma }_{{\mathrm{E}}}=18$ grid points. The excitatory coupling value is also shifted to demonstrate different states of the model, that is, ${W}_{{\mathrm{E}}}\,=\,0.2235\,+\,\Delta {W}_{{\mathrm{E}}}$.

As the neuron density is around 26516 mm^−2^ in cat primary visual cortex layer 2/3^67^ and the number of neurons in our model is 62500, the model contains approximately the same number of neurons as a 2.36 mm^2^ square patch of the primary visual cortex. Therefore, the distance between two neighboring neurons (one grid unit) in our circuit model is around 6.1 × 10^−3^ mm. In this study, we mainly use our model to account for why and how spontaneous activity is fundamentally related to stimulus-related response as found in visual cortex^2,5,10^ and ultimately to visual perception^15^. Because propagating activity patterns have been widely observed^12,13^ and because of the canonical nature of neural circuits across different brain areas^68^, the mechanism of computing by modulating spontaneous activity (CMSA) may be applicable for understanding cortical processing in other cortical areas as well.

In this study, we mainly focus on the network with the size of $250\,\times\,250$ ($N\,=\,250$); however, different sizes such as $N\,=\,150$, 200, and 300 are used to reveal the scaling behavior of the correlation length of pattern velocity fluctuations (Supplementary Fig. 4d–f). A random number generator produces random values for the initial membrane potentials from the resting (−75.625 mV) to the threshold potentials (−40 mV). Each trial excludes the first 1.5 s of transient time. Simulations use the Euler method with the time step of ${\mathrm{d}}t\,=\,0.1$ ms, using custom software written in C++.

### External inputs

To illustrate the computational mechanism of spontaneous activity, we find that it is necessary to use natural stimuli. In this study, we use face images, which are commonly used in psychophysical studies^15,69^. These images are processed with the Difference-of-Gaussian (DoG) filter (s.d. 1 and 2)^70,71^, which simulates the center-surround organization of the receptive field of retinal ganglion neurons that generated the feed-forward visual signal to V1^72,73^. The face image data set has 16 grayscale face images with neutral expression, normalized hairstyle, global orientation and lighting^69^. After the DoG filter, the images are resized to make their number of pixels equal the number of grid points in the network. All pixel values of the filtered image are normalized by their mean value and then multiplied by a scale parameter $S$ ($S=0.5$ nA, unless stated otherwise). These scaled pixel values correspond to the external inputs (i.e., ${I}_{{\mathrm{i}},{\mathrm{j}}}$ in Eq. 1) to the spiking network. After 2 s of spontaneous activity, the stimulus in input continuous until the simulation finishes; ${I}_{{\mathrm{i}},{{{\mathrm{j}}}}}\,=\,H(t-{t}_{{\mathrm{onset}}})\cdot {M}_{{\mathrm{i}},{\mathrm{j}}}$, where $H(\cdot )$ is the Heaviside function, $t$ is the time, ${t}_{{\mathrm{onset}}}=2$ s is the stimulus onset, and ${M}_{{\mathrm{i}},{\mathrm{j}}}$ is the value of pixel $({\mathrm{i}},{\mathrm{j}})$ of the scaled, resized and DoG filtered image.

### Tracking and characterizing propagating patterns

The spatiotemporal activity of our spatially extended network exhibits propagating wave patterns with complex dynamics. One of the salient features of these wave patterns is that they are localized, meaning that neurons that are firing in a certain interval are adjacent, and individual patterns are separated from each other. Based on this separation, we use an automatic method to identify these localized patterns as in our previous work^61^. We first choose a time window of duration 5 ms to detect enough spikes that are adjacent to each other, and we then use a flood-fill algorithm to classify groups of adjacent neurons as one pattern^74^. Two kinds of patterns emerge from our network model, patchy patterns and crescent-shaped waves (Fig. 1a). A significant difference between them is that the center of the former contains random firing activity, which results in gaps (i.e., holes) between firing neurons, whereas the latter does not. This property can be quantified by the Euler characteristic, which is the difference between the number of connected regions and the number of their holes^74^. For the crescent-shaped waves, the Euler characteristic is 1, and for the patchy patterns, it is $< {1}$. After identifying these propagating wave patterns automatically, we characterize their collective dynamics by calculating the center-of-mass (COM) position $({I}_{{\mathrm{M}}}(t),{J}_{{\mathrm{M}}}(t))$ of each pattern:

5 $${I}_{{\mathrm{M}}}(t)\,=\,\frac{1}{{M}_{{\mathrm{f}}}}\sum _{L=1}^{{M}_{{\mathrm{f}}}}{{\mathrm{i}}}_{{\mathrm{M}}}^{L}(t),\ {J}_{{\mathrm{M}}}(t)=\frac{1}{{M}_{{\mathrm{f}}}}\sum _{L=1}^{{M}_{{\mathrm{f}}}}{{\mathrm{j}}}_{{\mathrm{M}}}^{L}(t),$$

where ${{\mathrm{i}}}_{{\mathrm{M}}}^{L}(t)$ and ${{\mathrm{j}}}_{{\mathrm{M}}}^{L}(t)$ are the ${\mathrm{i}}$ and ${\mathrm{j}}$ positions of the $L$-th neuron that is firing at time $t$ in the $M$-th pattern, and ${M}_{{\mathrm{f}}}$ is the total number of firing neurons within this pattern.

To quantify the circular symmetry of the propagating patterns, we introduce the local order parameter $| \phi |$; for pattern ${\mathrm{j}}$, ${\phi }_{{\mathrm{j}}}$ is defined as

6 $${\phi }_{{\mathrm{j}}}=\frac{1}{{N}_{{\mathrm{j}}}}\sum _{k=1}^{{N}_{{\mathrm{j}}}}{e}^{{{{\mathrm{i}}}}{\theta }_{{\mathrm{j}}}^{{\mathrm{k}}}},$$

where ${N}_{{\mathrm{j}}}$ is the number of spikes in pattern ${\mathrm{j}}$, ${\mathrm{i}}=\sqrt{-1}$, and ${\theta }_{{\mathrm{j}}}^{k}$ is the azimuth of the $k$-th spiking neuron within pattern ${\mathrm{j}}$ with respect to a fixed axis and the center of mass of pattern ${\mathrm{j}}$. It enables us to quantify how activated elements of an individual pattern are organized around its center of mass. $| \phi |$ is small if all elements are roughly uniformly distributed around the center, whereas large $| \phi |$ indicates that activated elements are concentrated at a certain azimuth. Thus, crescent-shaped waves have larger $| \phi |$ than patchy patterns.

We also introduce another order parameter $\Phi$ for characterizing the collective motion of these patterns^13^.

7 $$\Phi =\frac{\parallel \sum _{{\mathrm{i}}=1}^{{{N}}}\vec{{{\bf{v}}}_{{\mathrm{i}}}}\parallel }{\sum _{i=1}^{{{N}}}\parallel \vec{{{\bf{v}}}_{{\mathrm{i}}}}\parallel },$$

where $N$ is the number of patterns, $\vec{{{\bf{v}}}_{i}}$ is the velocity of pattern ${\mathrm{i}}$. $\Phi$ ranges from zero to one, with $\Phi =1$ when velocity vectors align to one direction, reflecting directed coherent motion of propagating activity patterns across the neural network; if $\Phi$ is close to zero, it means that the motions of patterns are disordered, and that the velocities of the individual patterns point in random directions and average close to zero^13^.

To quantify the heterogeneity of pattern dynamics, we introduce an index

8 $$H=\frac{1}{T}\sum _{t=1}^{t=T}\frac{1}{{N}_{t}}\sum _{{\mathrm{i}}=1}^{{\mathrm{i}}={N}_{t}}{[{\nu }_{{\mathrm{i}}}(t)-\mu (t)]}^{2},$$

where ${\nu }_{{\mathrm{i}}}(t)$ is the speed of activity pattern $i$ at time $t$, $\mu (t)$ is the mean of pattern speeds at time $t$, ${N}_{t}$ is the number of patterns at time $t$, and $T$ is the total time.

### Cascade detection

The propagation of the local patterns gives rise to multiple spatiotemporally contiguous clusters resembling those found in ref. ^22^. We detect these contiguous clusters based on their spatiotemporal contiguity, with each of them being referred to as a cascade. Specifically, for each time step, connected components of spikes are clustered as an object within a radius ${r}_{{\mathrm{S}}}$, and a cascade is defined as a set of objects whose center of mass changes by less than ${r}_{{\mathrm{T}}}$ between successive time (1 ms resolution) (Fig. 1b). A cascade is quantified by two quantities: (i) size, i.e., the number of spikes within a cascade; (ii) duration, i.e., the number of successive time steps a cascade is active. In this study, we set ${r}_{{\mathrm{S}}}=1$ grid points, and ${r}_{{\mathrm{T}}}=5$ grid points; the results are not sensitive to these values. These cascades are spatially localized.

### Power-law fitting

Using maximum likelihood methods^23^, we fit power laws to the cascade distributions. The fitting function for the distribution of cascade size ($S$) and duration ($D$) is $f(X)={X}^{-\beta }{({\sum }_{x\,=\,{x}_{\min }}^{{x}_{\max }}{x}^{-\beta })}^{-1}$, where $f$ is the probability density function of $X$, ${x}_{\max }$ is the largest observed value of $X$ (no higher cutoff) and $X$ represents the fitted variable ($S$ or $D$). The lower bound ${x}_{\min }$ and the exponent $\beta$ are fitting parameters. ${x}_{\min }$ is fit in the interval of $[1,{x}_{\max }]$. 1 is the minimum observed size and duration. For each possible choice of ${x}_{\min }$, we estimate the exponent via the maximum likelihood method and the Kolmogorov–Smirnov (KS) test. We then select as our estimate of ${x}_{\min }$ (usually ${x}_{\min }< \, {3}$), the value that gives the minimum KS statistic over all values of ${x}_{\min }$. With the fixed ${x}_{\min }$, we then get the exponent giving minimum KS statistic. After finding the best-fit power law, we further assess the goodness-of-fit via log-likelihood ratio, and Vuong test.

### Dynamic range

The dynamic range is the range of stimulus intensities that can be processed by a network, and is often maximized near the criticality of a non-equilibrium phase transition^24,25^. After measuring responses (i.e., averaged firing rate, $R$) to a range of stimulus intensities ($S\in [{2}^{-3},{2}^{10}]$ nA, defined in External inputs section), we use the response curve, $R(S)$, to compute the dynamic range,

9 $$\Delta =10\,{\mathrm{log}}_{10}\left(\frac{{S}_{\max }}{{S}_{\min }}\right),$$

where ${S}_{\max }$ and ${S}_{\min }$ are the stimulus intensity leading to 90% and 10% of the range of $R$, respectively (Supplementary Fig. 3, left).

### Correlation length of velocity fluctuations of activity patterns

For each activity pattern, we can obtain its velocity based on Eq. 5. We next define a correlation function of the fluctuations of pattern velocities

10 $$C(r)=\frac{1}{{c}_{0}}\frac{\sum _{{\mathrm{i}},{\mathrm{j}}}\vec{{{\bf{u}}}_{{\mathrm{i}}}}\cdot \vec{{{\bf{u}}}_{{\mathrm{j}}}}\delta (r-{r}_{{\mathrm{i}},{\mathrm{j}}})}{\sum _{{\mathrm{i}},{\mathrm{j}}}\delta (r-{r}_{{\mathrm{i}},{\mathrm{j}}})},$$

where $\vec{{{\bf{u}}}_{i}}=\vec{{{\bf{v}}}_{i}}-\frac{1}{N}{\sum }_{k=1}^{N}\vec{{{\bf{v}}}_{k}}$, $\delta (r-{r}_{{\mathrm{i}},{\mathrm{j}}})$ is a Dirac $\delta$-function selecting pairs of patterns at mutual distance $r$, and ${c}_{0}$ is a normalization factor^27^. A large value of $C(r)$ indicates that the fluctuations are nearly parallel and thus strongly correlated. Conversely, when the fluctuations are antiparallel, and therefore anticorrelated, the correlation function has a negative value. In the state with regular waves only, all propagating waves have the same velocity (completely parallel), so $C(r)=0$. To increase the number pairs with the same $r$, we round the distance to the nearest tens digit, for example, $r=12.59\to 10$; otherwise, it is hard to get a meaningful average and normalize the correlation function. This correlation function $C(r)$, therefore, measures to what extent the velocity fluctuations are correlated (parallel). The function changes sign at $r=\xi$, which gives a good estimate of the average size of the correlated domains, and $\xi$ is defined as the correlation length (Supplementary Fig. 4a), as in ref. ^27^.

### Illustration of spatiotemporal coherent structures

To show the spatiotemporal co-activated patterns of spontaneous activity, we calculate the spike-triggered average (STA) of the smoothed membrane potential, given by

11 $${\mathrm{STA}}=\frac{1}{{n}_{{\mathrm{sp}}}}\sum _{{\mathrm{i}}=1}^{T}B({\mathrm{i}})F({\mathrm{i}}),$$

where $B({\mathrm{i}})$ is a binary function of a randomly picked neuron’s spiking activity (if this neuron fires at time step $i$, $B({\mathrm{i}})\,=\,1$, otherwise $B({\mathrm{i}})\,=\,0$), and ${n}_{{\mathrm{sp}}}={\sum }_{i=1}^{T}B({\mathrm{i}})$, the total number of spikes of this neuron. $F({\mathrm{i}})$ is the smoothed ${V}_{{\mathrm{m}}}$ frames, given by

12 $$F({\mathrm{i}})=[{V}_{{\mathrm{m}}}({\mathrm{i}})-\overline{{V}_{{\mathrm{m}}}({\mathrm{i}})}]* G,$$

where ${V}_{{\mathrm{m}}}(i)$ is the membrane potential of all neurons at time step $i$, the bar represents the mean across all neurons, $*$ is the convolution operation with periodic boundary conditions, and $G$ is the Gaussian filter (s.d.: 25).

To characterize the spatial correlation of the activity patterns emerging in our model, we calculate the correlation map $C(s,x)={\mathrm{corr}}(f(s),f(x))$, where $f(\ldots)$ is the instantaneous firing rate of a neuron obtained by a 250 ms bin and sliding over in 250-ms steps (Supplementary Fig. 5c), $s$ is the seed neuron, $x$ represents other neurons, and ${\mathrm{corr}}$ represents Pearson correlation over 2 s^7^. By computing $C(s,x)$ for all the other neurons $x$ in the 2D network, a 2D correlation map with respect to the seed neuron can be obtained. The fracture is defined as the rate by which the correlation pattern changes when changing the seed point location over one grid point^7^:

13 $$F(s)=\sqrt{{F}_{{\mathrm{dx}}}{(s)}^{2}+{F}_{{\mathrm{dy}}}{(s)}^{2}},$$

where ${F}_{{\mathrm{dx}}}(s)\ ({F}_{{\mathrm{dy}}}(s))$ signifies the $x\ (y)$-component of the rate of change of the correlation pattern at seed point $s$. We approximate this rate of change by the (second-order) correlation between two correlation patterns with the seed points at adjacent pixels one grid point apart:

14 $${F}_{{\mathrm{dx}}}(s)=1-{C}_{{\mathrm{dx}}}(s),$$

15 $${C}_{{\mathrm{dx}}}(s)={{\mathrm{corr}}}_{x}(C(s,x),C(s+{1}_{x},x)),$$

where ${{\mathrm{corr}}}_{x}(\cdots \ )$ denotes the Pearson correlation coefficient calculated over all locations $x$ and ${1}_{x}$ is one grid point in the $x$-direction.

### Indices for quantifying the modulation processes

To quantify the modulation processes of spontaneous activity patterns caused by eternal inputs, we introduce two indices. The first one is based on the change of the geometrical property of these propagating patterns before and after the stimulus onset; that is, during the modulation processes, the shape of the localized patterns can change from a crescent to a circle. We thus use the order parameter defined in Eq. 6 to introduce a modulation index:

16 $$\eta =\frac{{\left\langle \bar{| \phi | }\right\rangle }_{{\mathrm{spon}}}-{\left\langle \bar{| \phi | }\right\rangle }_{{{\mathrm{t}}}_{1}-{{\mathrm{t}}}_{2}}}{{\left\langle \bar{| \phi | }\right\rangle }_{{\mathrm{spon}}}+{\left\langle \bar{| \phi | }\right\rangle }_{{{\mathrm{t}}}_{1}-{{\mathrm{t}}}_{2}}},$$

where $| \phi |$ is the averaged order parameter norm across all patterns, $\left\langle \ldots \right\rangle$ denotes averaging over time, and the bar represents averaging over patterns; ${t}_{1}=25$ ms, and ${t}_{2}=75$ ms (0 ms is set as the stimulus onset). These specific values of ${t}_{1}$ and ${t}_{2}$ are chosen because crescent-shape wave patterns normally change to patchy patterns from ${t}_{1}$ to ${t}_{2}$. The same bin size of 50 ms is used to average the order parameter of spontaneous activity patterns. Another modulation index is based on the averaged population firing rate, defined as

17 $$\zeta =\frac{{F}_{{\mathrm{spon}}}-{F}_{{\mathrm{evok}}}}{{F}_{{\mathrm{spon}}}+{F}_{{\mathrm{evok}}}},$$

where ${F}_{{\mathrm{spon}}}$ and ${F}_{{\mathrm{evok}}}$ are the averaged firing rates of all neurons 250 ms before and after the stimulus onset, respectively; the results are not sensitive to the bin size as long as it is larger than 120 ms (modulation time).

### Mean-matched Fano factor

The Fano factor (FF) measures the trial-to-trial variability of the spike count ${n}_{{\mathrm{r}}}(t-\frac{\Delta t}{2},t+\frac{\Delta t}{2})$; $t-\frac{\Delta t}{2}$ and $t+\frac{\Delta t}{2}$ indicate the start and end points, respectively, of the time window of size $\Delta t$ over which the spikes are counted. $\Delta t=250$ ms, and the calculation slides over in 10-ms steps unless stated otherwise. The FF of a neuron at $r=({\mathrm{i}},{\mathrm{j}})$ is then:

18 $${{\mathrm{FF}}}_{{\mathrm{r}}}(t)=\frac{{\mathrm{var}}[{n}_{{\mathrm{r}}}(t-\frac{\Delta t}{2},t+\frac{\Delta t}{2})]}{\bar{{n}_{{\mathrm{r}}}}(t-\frac{\Delta t}{2},t+\frac{\Delta t}{2})},$$

where $\bar{{n}_{{\mathrm{r}}}}(t-\frac{\Delta t}{2},t+\frac{\Delta t}{2})$ and ${\mathrm{var}}[{n}_{{\mathrm{r}}}(t-\frac{\Delta t}{2},t+\frac{\Delta t}{2})]$ are the mean and variance, respectively, of the spike count across repeated trials with random initial conditions. Note that the FF of a homogeneous (with a constant rate of events) Poisson process is 1, whereas that of a periodic process is 0. As in ref. ^30^, we use mean-matching to calculate the FF; this ensures that a declining FF is not trivially related to rising rates (e.g., due to an increase in the size of the denominator in Eq. 18). The mean-matching method finds a common distribution of firing rates across all time steps, and ignores data points that do not fit this distribution^30^. To calculate this common distribution, we organize firing rates into histogram bins, which we choose to be of width 10 Hz.

### Bubbles and comparison with evoked patterns

In psychophysical studies^15^, it has been found that a face mask punctured by sparse, randomly located Gaussian windows called perceptual bubbles reveal enough information to correctly recognize faces. As in ref. ^15^, we use an ideal observer to identify these perceptual bubbles. It has three major steps (Supplementary Fig. 12). First, a square plane is randomly punctured by 2D-Gaussian windows. These Gaussian windows have a fixed standard deviation, ${D}^{{\mathrm{E}}}$ which is the neural coupling range in our spiking circuit model. The number of these bubbles, $15\pm 2$, is approximately the same as the number of evoked patterns in our circuit model. Such a punctured plane is a mask. Second, every mask is put on 16 processed face images by multiplying the mask and a processed face image element-wise. Third, we calculate the mean 2D Pearson correlation of all pairs of masked face images; for instance, 16 face images have 120 pairs. The process is repeated $1{0}^{6}$ times to generate $1{0}^{6}$ masks and compare the mean 2D correlations. The mask that has a minimum mean correlation is denoted the best bubble mask because it reveals the areas with the highest local variances that contain more information^15^. To compare the best bubble mask with evoked patterns, we convert the evoked activity patterns to 2D Gaussian located at the pattern centers of mass with ${\mathrm{s.d.}}={D}^{{\mathrm{E}}}$ (Fig. 7c), referred to as a 2D converted map. In the converting process, activity patterns outside the face are removed because they are not directly relevant to face-image-evoked responses. The similarity between the 2D converted maps and the best bubble mask is quantified by the 2D Pearson correlation.

### Population decoding analysis

The acceleration of response by spontaneous activity is assessed through a decoding analysis^37^. A multi-class classifier is used to assess the information about the stimuli encoded in the neural activity under two conditions: the CMSA and the control cases. In the CMSA case, stimuli are added after 2 s of spontaneous activity; in the control cases, the spontaneous membrane potentials are randomly shuffled among neurons prior to stimulus onset. The input data are constructed from the summed peristimulus time histogram (PSTH) of 100 neurons (in each bin, the spike count is the sum of 100 neurons). The same number of trials across stimuli and the conditions are used. The 100 neurons are randomly sampled from the neurons exposed by the best bubbles selected by the ideal observer (eyes, mouth, etc), namely, the sampled neurons really carry the information of stimuli. The summed PSTH is collected in the bin of 200 ms and slid over in 50-ms steps. The PSTH of all 16 face-image stimuli in each condition is split into training and test sets for cross-validation (leave-one-out). From the training set, we then create bootstrapped sub-training sets ${L}_{b}$, for $b=1,\cdots \ ,B$, where $B=10$, by sampling with replacement from $L$ (the fraction of unique examples in ${L}_{b}$ is $1-\frac{1}{{\mathrm{e}}}$, where ${\mathrm{e}}=2.71828\cdots$)^75^. Templates are created for each stimulus, condition, and time bin in each sub-training set ${L}_{b}$ by averaging the spike counts among all trials in each bin. Summed spike counts for each test trial are classified according to the smallest Euclidean distance from the templates across *B* bootstrapped sub-training sets in each bin, obtaining *B* different votes for each bin. Decoding accuracy in a given bin is defined as the fraction of correctly classified test trials in that bin.

The significance of decoding accuracy is established via a permutation test: 1000 shuffled datasets are created by randomly permuting stimulus labels among trials, and a shuffled distribution of 1000 decoding accuracies is obtained. In each bin, decoding accuracy of the original dataset is deemed significant if it exceeds the upper boundary, ${{\alpha}}_{{0.05}}$, of the 95% confidence interval of the shuffled accuracy distribution in that bin (this includes a Bonferroni correction for multiple bins, so that ${\alpha}_{0.05}={1-0.05}/{N}_{{\mathrm{b}}}$, with ${N}_{{\mathrm{b}}}$ the number of the number of bins). Decoding latency is estimated as the earliest bin with significant decoding accuracy.

### Reporting summary

Further information on research design is available in the Nature Research Reporting Summary linked to this article.

## Supplementary information

Supplementary Information

Description of Additional Supplementary Files

Supplementary Movie 1

Supplementary Movie 2

Supplementary Movie 3

Reporting Summary

Source Data

## Data Availability

All data are available from the corresponding authors upon request. The source data underlying Figs. 1c, d, f, 2b, d, 3f, g, 4–6, and 7e and Supplementary Figs. 2, 3, 4a–c, 8, 9, 11, 13e, 14b–e, and 15 are provided as a Source Data file.

## Code availability

Custom C++ code is available at https://github.com/BrainDynamicsUSYD/SpikeNet.
