# Supplementary material for: Computing by modulating spontaneous cortical activity patterns as a mechanism of active visual processing
Source: Nat Commun. 2019 Oct 29;10:4915. doi: 10.1038/s41467-019-12918-8 (PMC6820766; doi:10.1038/s41467-019-12918-8)
Supplement: Supplementary file 1 — Supplementary Information [file 41467_2019_12918_MOESM1_ESM.pdf]

Supplementary Information for:

Computing by modulating spontaneous cortical activity patterns as a mechanism of active visual processing

Chen et al.

Supplementary Note

- The CMSA mechanism for different natural stimuli and in different models.
- Analysis of the CMSA process.

Supplementary Figures and Legends

Supplementary Figure 1. Shifting the excitatory coupling value ΔW_{E} of the network gives rise to ordered and disordered activity states, and the modulation effect is maximized near the critical transitions state.

Supplementary Figure 2. The distributions of cascade size and duration in non-critical states cannot be fitted as power-law functions.

Supplementary Figure 3. Stimulus-response curves and dynamic range Δ.

Supplementary Figure 4. The correlation length is maximized and scales linearly with the size of the network in the critical regime.

Supplementary Figure 5. Time series of spike count shows intermittent fluctuations.

Supplementary Figure 6. Spike-triggered averaged (STA) membrane potentials (V_{m}) patterns show regular shapes when the network is shifted away from the critical regime, and the STA-V_{m} patterns are similar for the network with different initial conditions in the critical regime.

Supplementary Figure 7. Snapshots of membrane potential for a sub-network (66×66 grid points) after stimulus onset illustrate the modulation process of a spontaneous activity pattern.

Supplementary Figure 8. Stimulus onset quenches neural variability.

Supplementary Figure 9. Decoding latency and accuracy as a function of ΔW_{E}.

Supplementary Figure 10. Shuffling spontaneous membrane potentials eliminates their correlations with input-related responses.

Supplementary Figure 11. In the non-critical regimes, correlations between spontaneous and evoked activities are weak.

Supplementary Figure 12. The schematic diagram of perceptual bubbles and the method of how the ideal observer generates the best bubble mask.

Supplementary Figure 13. The similarity of the bubbles selected by the ideal observer and the evoked activity patterns is maximized in the critical regime.

Supplementary Figure 14. The effect of noise on the CMSA mechanism.

Supplementary Figure 15. The firing rate and the distance between neighboring patterns change as a function of the coupling range, D^{λ}.

Supplementary Figure 16. CMSA for different natural images.

Supplementary Figure 17. In the firing rate model, spontaneous activity patterns are modulated by face images.

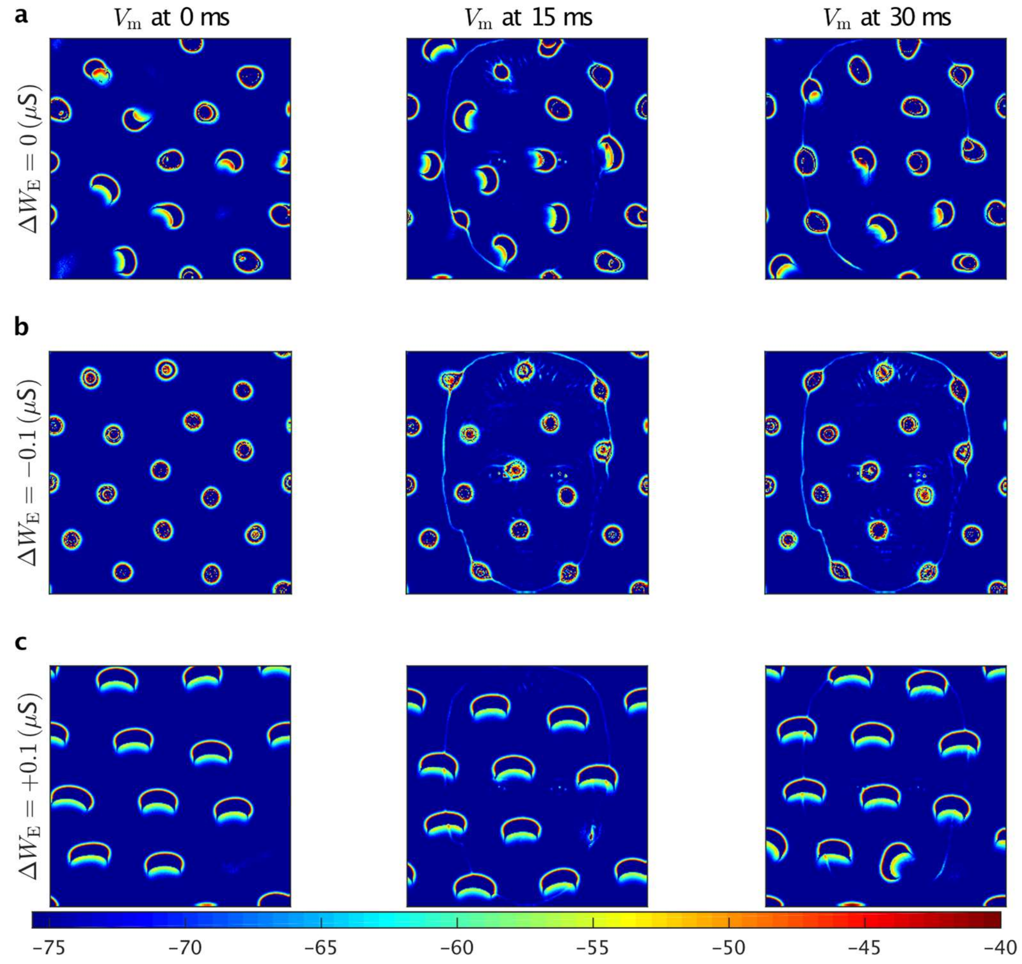

Supplementary Figure 1. Shifting the excitatory coupling value ΔW_E of the network gives rise to ordered and disordered activity states, and the modulation effect is maximized near the critical transitions state. a Snapshots of membrane potentials in the network at 0, 15, 30 ms when the network is close to the critical point. **b** Same as in **a** but for the inhibition dominant case ($\Delta W_E = -0.1\ \mu$S). **c** Same as in **a** but for the excitation dominant case ($\Delta W_E = 0.1\ \mu$S). The spontaneous patterns in **b** and **c** are not trapped by stimuli. 0 ms is the stimulus onset. Color represents membrane potential.

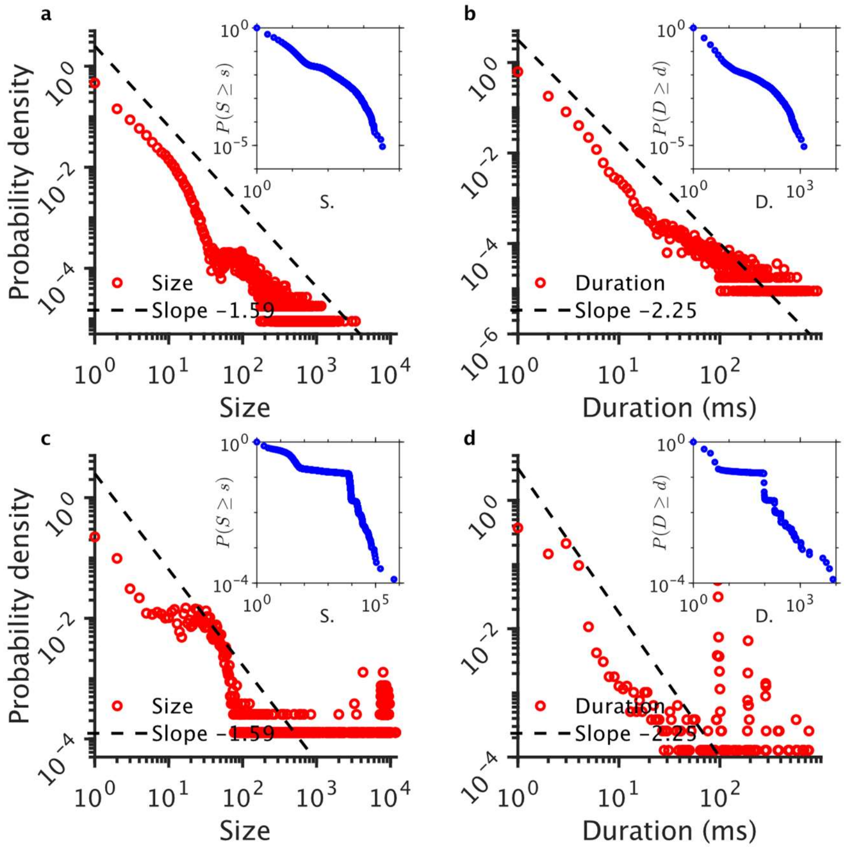

Supplementary Figure 2. The distributions of cascade size and duration in non-critical states cannot be fitted as power-law functions. a The distribution of cascade size cannot be fitted as a power-law function when $\Delta W_{\mathrm{E}} = -0.1$ μS. Inset: the complementary cumulative distribution function of the same data also does not follow a power-law function. **b** Same as in **a** but for duration distribution. **c-d** Same as in **a-b**, but for $\Delta W_{\mathrm{E}} = 0.1$ μS. These distributions cannot pass the Kolmogorov-Smirnov test.

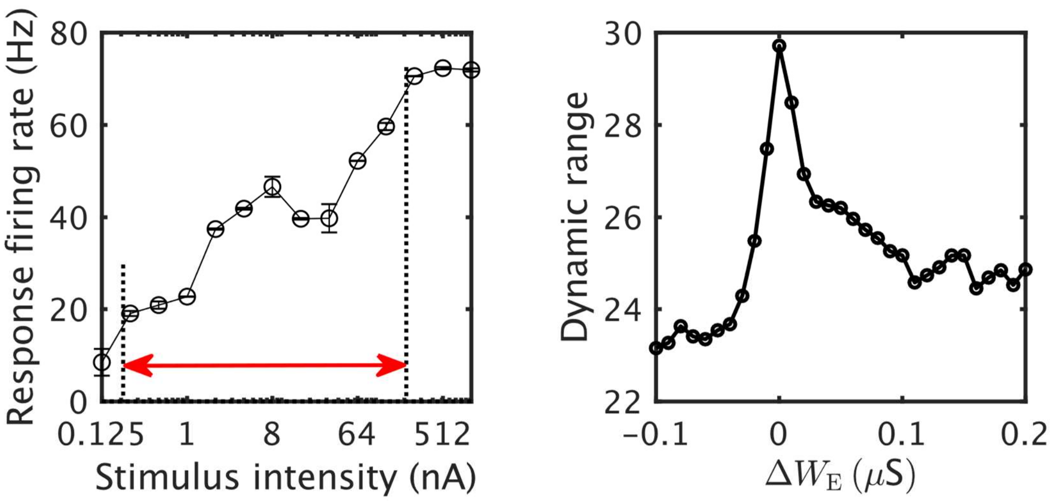

Supplementary Figure 3. Stimulus-response curves and dynamic range $\mathit{\Delta}$. Left: The response of networks (firing rate) evoked by stimuli with different intensity values in the critical regime ($\mathit{\Delta} W_{\mathrm{E}} = 0\ \mu$S). The two dashed lines label the stimulation intensity leading to 10% and 90% of the range of the response firing rate. The red arrow denotes the dynamic range. The response firing rates average over 10 trails with random initial conditions. Right: Dynamic range $\mathit{\Delta}$ as a function of $\mathit{\Delta} W_{\mathrm{E}}$. This indicates that the dynamic range is maximal in the critical regime ($\mathit{\Delta} W_{\mathrm{E}} = 0\ \mu$S). Source data are provided as a Source Data file.

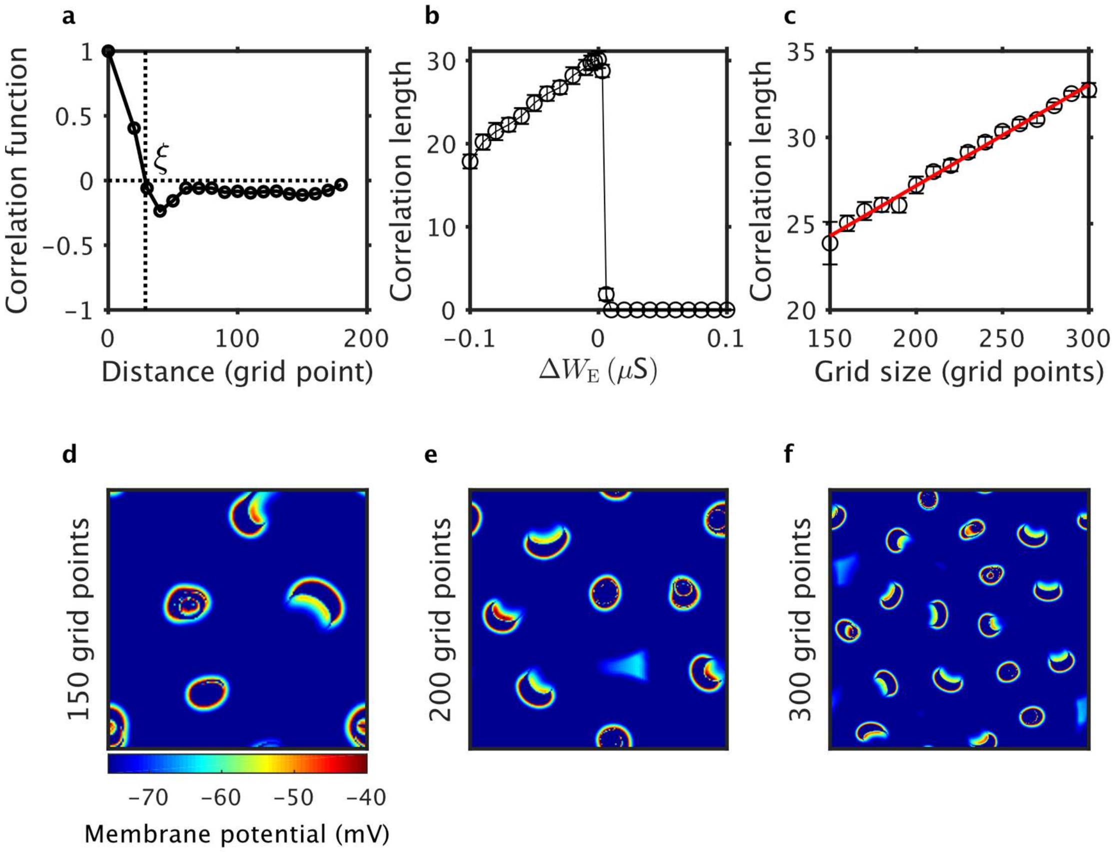

Supplementary Figure 4. The correlation length is maximized and scales linearly with the size of the network in the critical regime. a The correlation of the fluctuations of pattern velocities as a function of the distance between activity patterns in the critical regime ($\Delta W_{\mathrm{E}} = 0\ \mu$S). The correlation length ξ is defined as the distance where correlation changes sign. **b** The correlation length is maximized in the critical regime, $\Delta W_{\mathrm{E}} = 0\ \mu$S. The correlation length is 0 when $\Delta W_{\mathrm{E}} > 0\ \mu$S, because in the regular wave regime, localized crescent wave patterns propagate without fluctuations. Error bars are s.e.m.. The correlation lengths are obtained by averaging over 45 trials with random initial conditions. **c** The correlation length ξ as a function of the linear grid size of the networks. Error bars are s.e.m.. The correlation length grows linearly with the network size, signaling the presence of scale-free correlation length and criticality. Error bars are s.e.m.. Each point is obtained by averaging over 50 trials with random initial conditions. Source data are provided as a Source Data file. **d-e** The snapshots of membrane potentials in the networks with the size of 150, 200, and 300, respectively, show similar dynamical patterns as in the network with the size of 250.

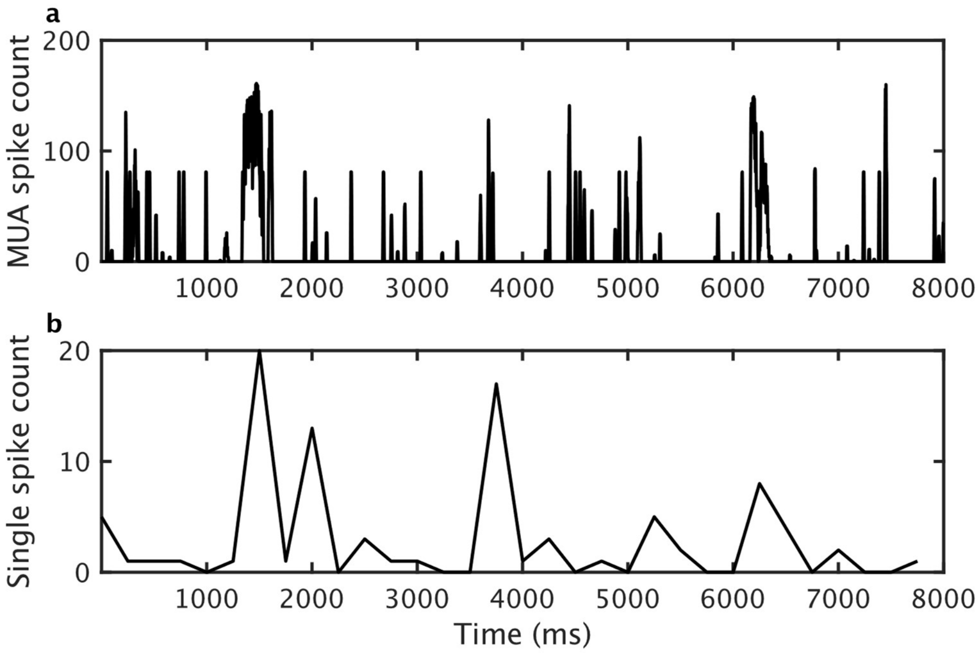

Supplementary Figure 5. Time series of spike count shows intermittent fluctuations. a The time series of multi-unit activity spike count of a randomly chosen electrode (10 ms bin, sliding over in 1-ms steps. Multi-unit activity is defined as the summed activity of a local group of neurons (81 neurons within a circle with 5-grid-point radius). **b** Time series of single-neuron spike count (250 ms bin, sliding over in 250-ms steps, i.e., no overlapping). The same time series is used to calculate the correlation map shown in Fig. 2a.

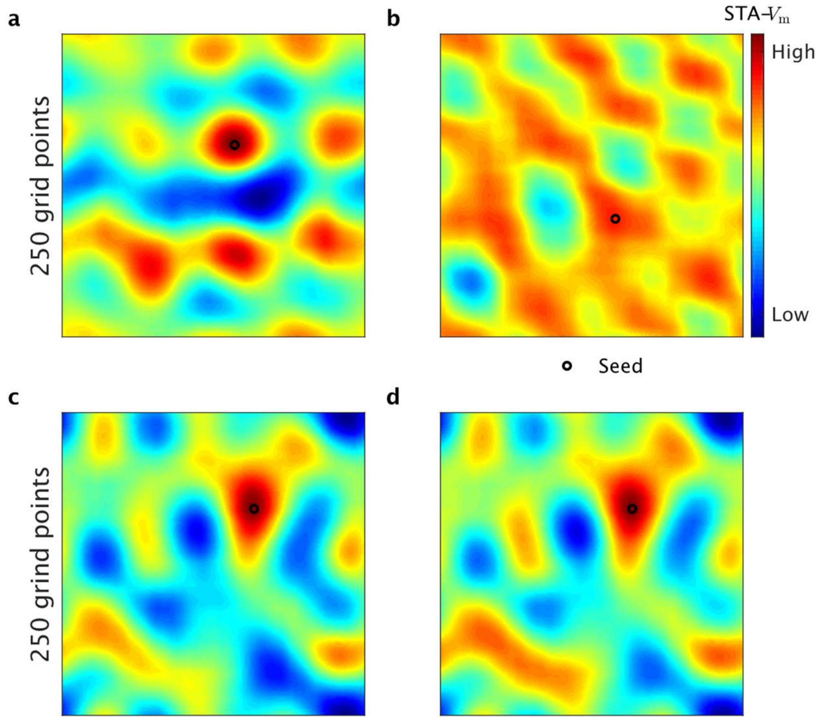

Supplementary Figure 6. Spike-triggered averaged (STA) membrane potentials (V_m) patterns show regular shapes when the network is shifted away from the critical regime, and the STA-V_m patterns are similar for the network with different initial conditions in the critical regime. a STA-V_m shows regular shape in the state with patchy patterns only. It is calculated over 5 s. **b** Same as in **c** but when the state is shifted to regular wave sate. **c, d** The same seed neuron with different initial conditions produces the similar spatial structure of STA-V_m pattern in the critical regime. These STA-V_ms are calculated over 5 s. The black circle labels the seed neuron.

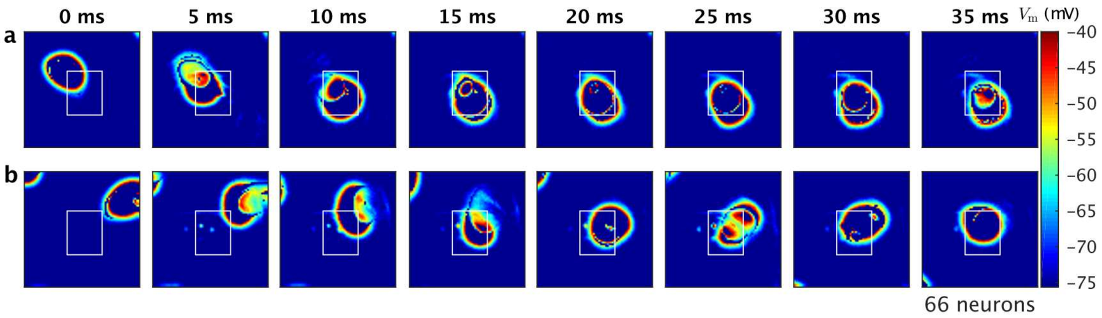

Supplementary Figure 7. Snapshots of membrane potential for a sub-network (66×66 grid points) after stimulus onset illustrate the modulation process of a spontaneous activity pattern. a An example of the stimulus (labeled by the white box) modulates the spontaneous patchy pattern. Column 1 starts at 0 ms (stimulus onset), and the following columns show sampled membrane potential (66×66 grid points) in every 5 ms. **b** Same as in **a** but for a crescent wave.

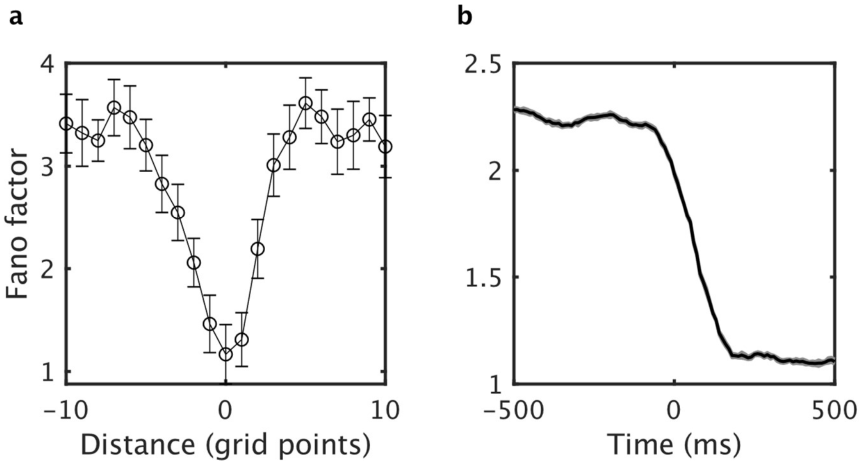

Supplementary Figure 8. Stimulus onset quenches neural variability. a An M-shaped spatial structure of Fano factor (±s.d. over 10 trials) as a function of the distance from the RoIs (region of interest, such as eyes and noses). **b** Fano factor (shaded area denoting s.e.m. among 10 trials) as a function of time. Fano factor is calculated with 250 ms bin. 0 ms is the stimulus onset time. Source data are provided as a Source Data file.

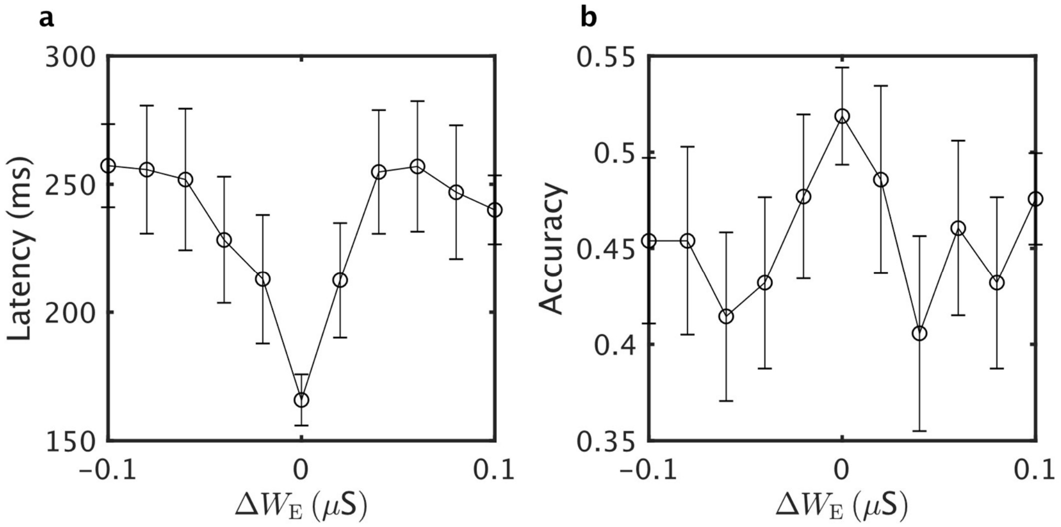

Supplementary Figure 9. Decoding latency and accuracy as a function of ΔW_{E}. a The decoding latency versus ΔW_{E}. The decoding latency is the shortest in the critical regime ($\Delta W_{\mathrm{E}} = 0\ \mu$S), indicating the response is fastest in this regime. **b** The decoding accuracy at 100 ms latency as a function of ΔW_{E}. The decoding accuracy at 100 ms is the highest in the critical regime ($\Delta W_{\mathrm{E}} = 0\ \mu$S). The error bars represent s.e.m.. Source data are provided as a Source Data file.

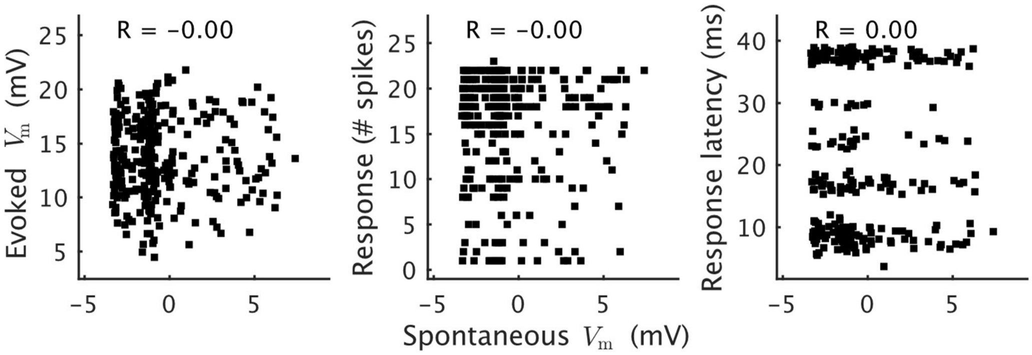

Supplementary Figure 10. **Shuffling spontaneous membrane potentials eliminates their correlations with input-related responses. Left:** Scatter plot of the mean V_m of the evoked response over a period of T (2-120 ms, mean value: 8 ms) after the stimulus onset versus the shuffled spontaneous mean V_m over T immediately before the stimulus onset. T is the duration of the modulation process, that is, the time interval from the stimulus onset to the time when the spontaneous pattern is trapped. **Middle:** Scatter plot of the number of spikes occurring in the first $2T$ of the response and the shuffled mean spontaneous V_m in the T preceding the stimulus onset. **Right:** Scatter plot of latency to the first spike and the shuffled spontaneous V_m. Calculations are performed as for Fig. 6a-c.

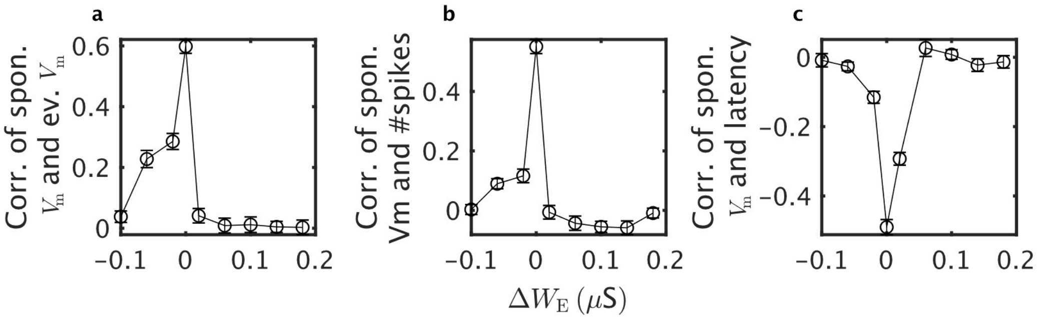

Supplementary Figure 11. In the non-critical regimes, correlations between spontaneous and evoked activity are weak. a Correlation between averaged spontaneous and evoked membrane potentials in 64 ms as a function of ΔW_E. **b** Correlation between averaged spontaneous membrane potential in 64 ms and the number of spikes in the first 128 ms of the response as a function of ΔW_E. **c** Correlation between averaged spontaneous membrane potential in 64 ms and the latency of the first spike in the first 128 ms of the response as a function of ΔW_E. Corr., correlation, spon., spontaneous, ev., evoked. Error bars represent s.d., calculated among 10 trials at each ΔW_E value. Source data are provided as a Source Data file.

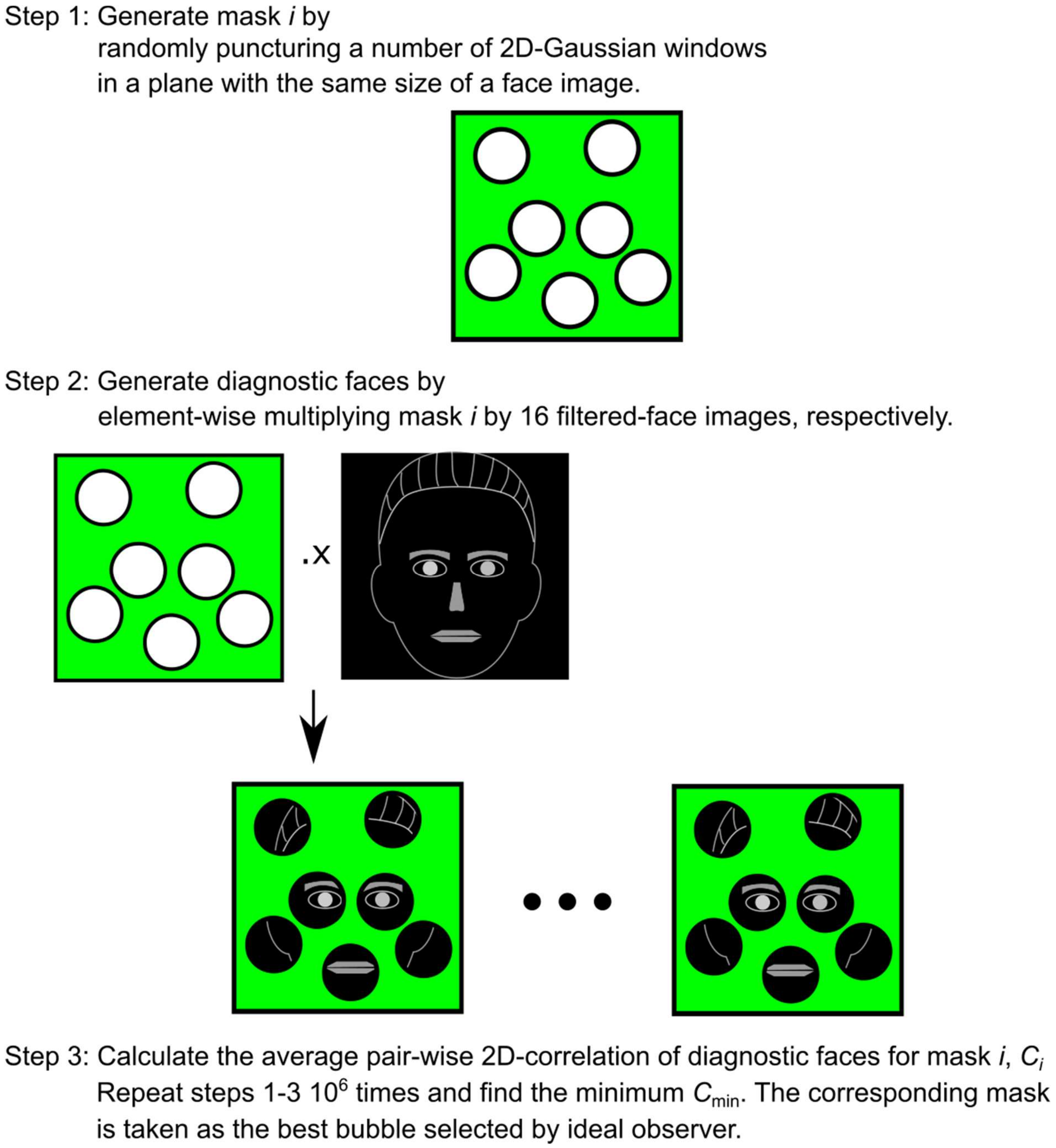

Supplementary Figure 12. Schematic diagram of perceptual bubbles and the generation of the best bubble selected by ideal observer. The cartoon faces represent the filtered-face images.

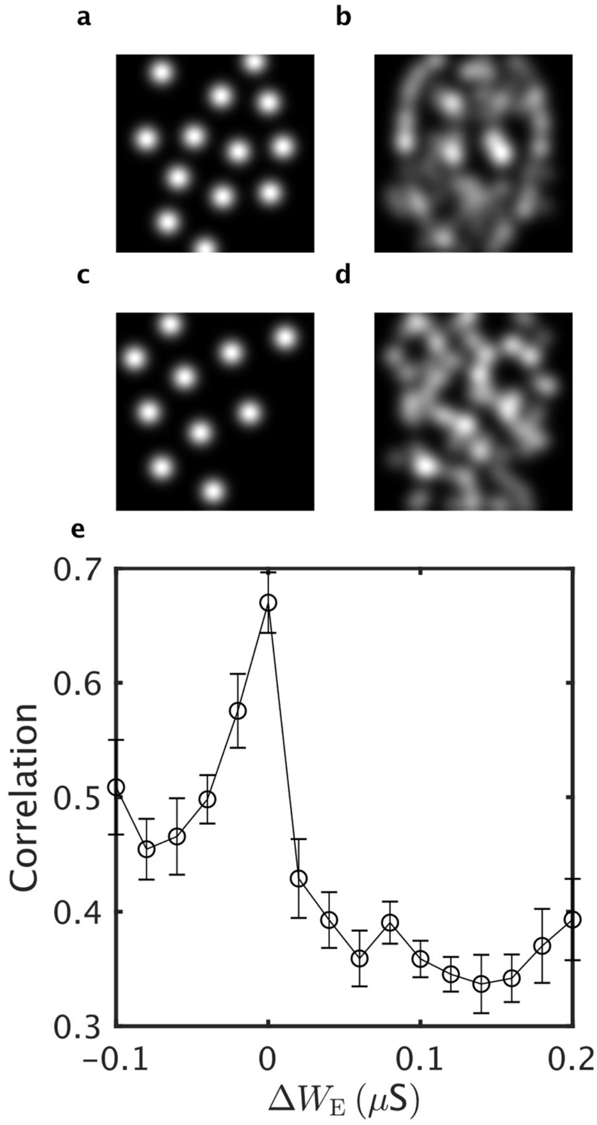

Supplementary Figure 13. The similarity of the bubbles selected by the ideal observer and the evoked activity patterns is maximized in the critical regime. a The 2D converted map of modulated patterns in a single trial for $\varDelta W_{\mathrm{E}} = -0.1$ μS. **b** The averaged 2D converted map of modulated patterns over 10 trials for $\varDelta W_{\mathrm{E}} = -0.1$ μS. **c** Same as in **a** but for $\varDelta W_{\mathrm{E}} = 0.1$ μS. **d** Same as in **b** but for $\varDelta W_{\mathrm{E}} = 0.1$ μS. **e** The correlation between the best bubbles selected by the ideal observer and 2D converted maps is maximum in the critical regime. Error bars represent s.e.m., calculated among 10 trials at each $\varDelta W_{\mathrm{E}}$ value. Source data are provided as a Source Data file.

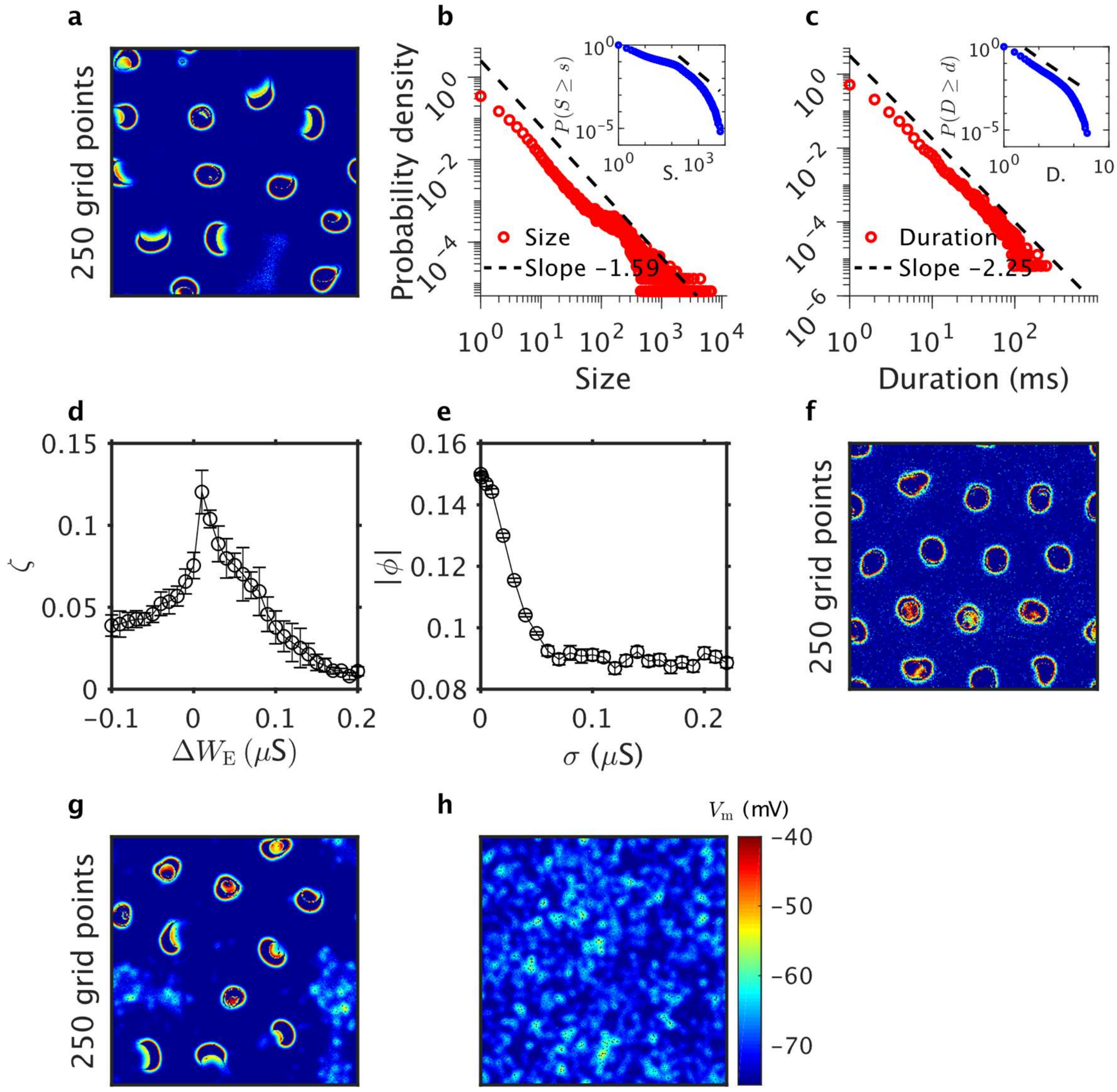

Supplementary Figure 14. The effect of noise on the CMSA mechanism. a The snapshot of membrane potentials when noise intensity $\sigma = 0.02\ \mu$S still can show dynamical patterns. **b** The distribution of cascade size follows a power-law function when $\sigma = 0.02\ \mu$S. Inset: the complementary cumulative distribution function of the same data also follows a power-law function. **c** Same as in **b** but for duration distribution. **d** The modulation index of firing rates (ζ) versus the excitatory coupling changes (ΔW_{E}) in the network with Gaussian noise $\sigma = 0.02\ \mu$S. ζ also has a maximum value in the critical regime. The error bars represent s.e.m.. **e** The local order parameter of activity pattern shape decreases as σ increases. Error bars are s.e.m.. **f** The snapshot of membrane potentials when $\sigma = 0.12\ \mu$S only shows patchy patterns. **g** The snapshot of membrane potentials when Poisson spikes are added to every neuron in the network with a rate of 1 Hz. **h** Same as in **g**, but with a rate of 5 Hz.

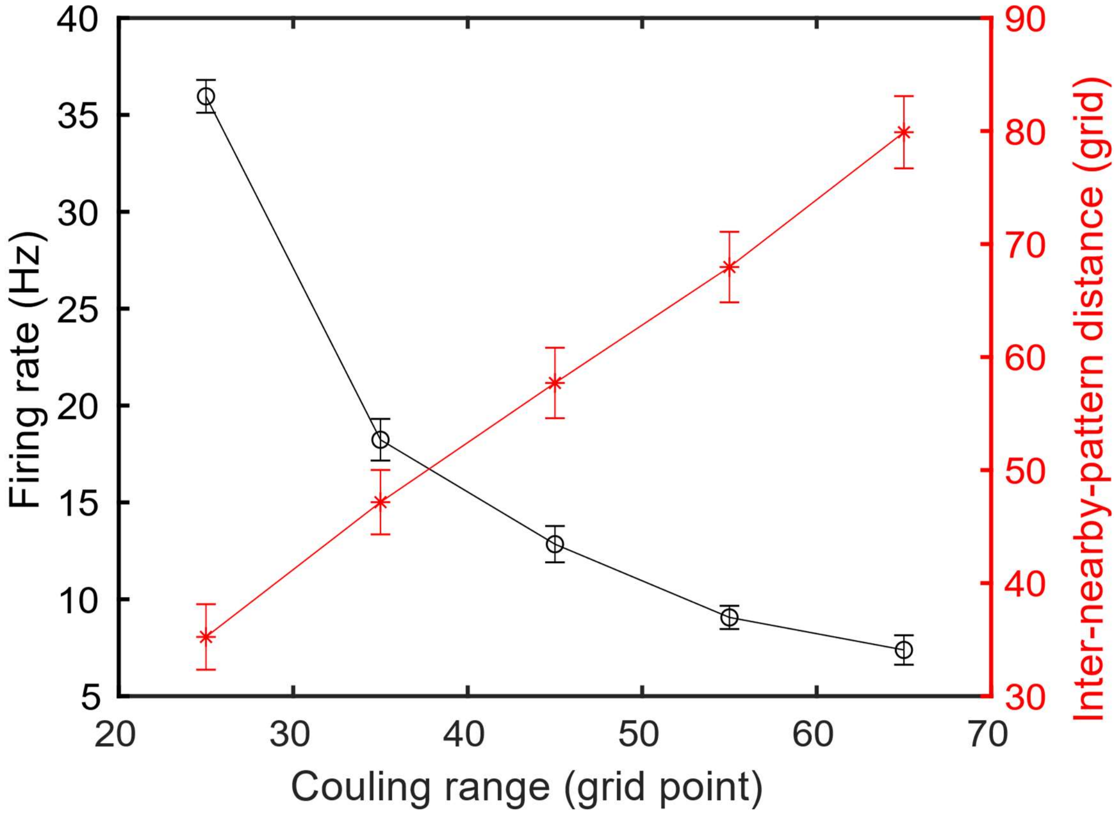

Supplementary Figure 15. **The firing rate and the distance between neighboring patterns change as a function of the coupling range, D^{λ}.** As D^{λ} increases, the number of patterns and firing rate decreases, and the distance between nearby patterns increases. Although the distance between neighboring patterns exists, the patterns can visit all space of the network because of the complex dynamics. Error bars are s.d.. Source data are provided as a Source Data file.

Supplementary Note 1

CMSA for different natural stimuli. We show the modulation process in the exponential leaky integrate-and-fire model evoked by different natural images such as an chrysanthemum (Supplementary Figure 16a). Following the same procedure as done for human face images, we filter the image by DoG (Supplementary Figure 16b) and add it to the neural network as external inputs. After the stimulus onset, this stimulus also modulates the spontaneous activity patterns (Supplementary Figure 16c-f).

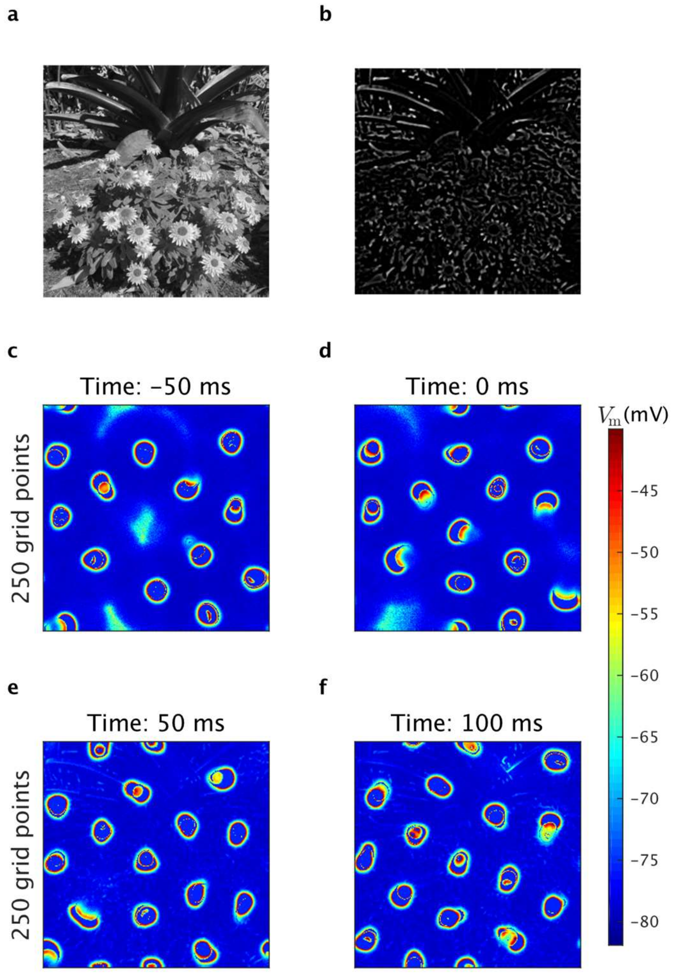

Supplementary Figure 16. CMSA for different natural images. a A image of chrysanthemum. **b** The filtered natural image by DoG. **c** Snapshot of membrane potential activity patterns generated by the spiking circuit with the filtered natural image at 50 ms before the stimulus onset. **d** Same as **c** but at the stimulus onset. **e** Snapshot of membrane potential at 50 ms after the stimulus onset. The activity patterns are redistributed and modulated by the saliency area of the nature image. **f** Same as **e** but at 100 ms after the onset. Color in **c-f**: membrane potential (mV).

CMSA in different models. To verify that the CMSA mechanism is not restricted to our particular choice of neural circuit models, we use a firing rate model[1]. This model also has a critical phase transition point between regular propagating waves and patchy patterns[1]. This 2D firing rate model is described as follows:

$$\begin{cases} \dfrac{\partial f}{\partial t} = -f + (1 - f - h)H(s - \kappa), & (1) \\ \dfrac{\partial h}{\partial t} = -ph + f, & (2) \end{cases}$$

where $f(x, y, t)$ and $h(x, y, t)$ represent the fraction of neurons that are in the firing and refractory states, respectively; the resting neurons' fraction $j(x, y, t)$ is $j = 1 - f - h$. In Eq. 1, $\kappa = 1$ is the firing threshold; $p = 0.30$ is a control parameter, which is chosen to ensure that the spontaneous dynamics of the rate model are the same as that of the spiking circuit model; and $H(\cdot)$ is a Heaviside function. The input $s(x, y, t)$ is given by

$$s(x, y, t) = u(x, y, t) + g(x, y, t), \qquad (3)$$

where $u(x, y, t) = w * f$ is the synaptic input, $*$ indicates a spatial convolution, w is the neural coupling strength, and $g(x, y, t)$ is the external stimulus (i.e. DoG-filtered face images), similar to that applied in the spiking circuit model. w is described as:

$$w(r) = W_{\mathrm{E}} w_K(r/\sigma_{\mathrm{E}}) - W_I w_k(r/\sigma_{\mathrm{I}}), \qquad (4)$$

where $r = \sqrt{x^2 + y^2}$ is the Euclidean distance, and $w_K(r) = \frac{2}{3\pi}[K_0(r) - K_0(2r)]$ (K_ν is the modified Bessel function of the second kind of order ν) and the subscripts E and I denote excitatory and inhibitory coupling, respectively. The function $w_K(r)$ approximates the exponential function $\mathrm{e}^{-r}/2\pi$ and it is normalized i.e. $\int w_K(r)dr = 1$. To ensure that the firing rate model has the same ratio of balance of excitation and inhibition as in the spiking circuit model, we first measure the excitatory synaptic currents $C_{\mathrm{E}}(t) = g_{\mathrm{E}}(t)[V(t) - V_{\mathrm{E}}]$ and inhibitory synaptic currents $C_{\mathrm{I}}(t) = g_{\mathrm{I}}(t)[V(t) - V_{\mathrm{I}}]$ of each neuron in the spiking circuit model. We then calculate the ratio $\beta = \langle \overline{C}_{\mathrm{E}} \rangle / \langle \overline{C}_{\mathrm{I}} \rangle$ where $\langle \cdot \rangle$ indicates averaging over time and the bar indicates averaging across neurons. The connectivity parameters of the firing rate model are then chosen to guarantee that the excitatory and inhibitory areas of $w(r)$ have the same ratio as β; the resultant parameters are: $W_{\mathrm{E}} = 910.51$, $W_{\mathrm{I}} = 420.98$, $\sigma_{\mathrm{E}} = 1.87$, and $\sigma_{\mathrm{I}} = 3.24$.

The spontaneous activity of the firing rate model exhibits propagating waves with complex dynamic, resembling the patterns emerging in the spiking neural circuit (Supplementary Figure 17a, b). After the onset of stimuli, the ongoing propagating patterns are modulated or redistributed to the salient region of stimulus and become

patchy patterns (Supplementary Figure 17c, d), consistent with the phenomena shown in our spiking circuit model.

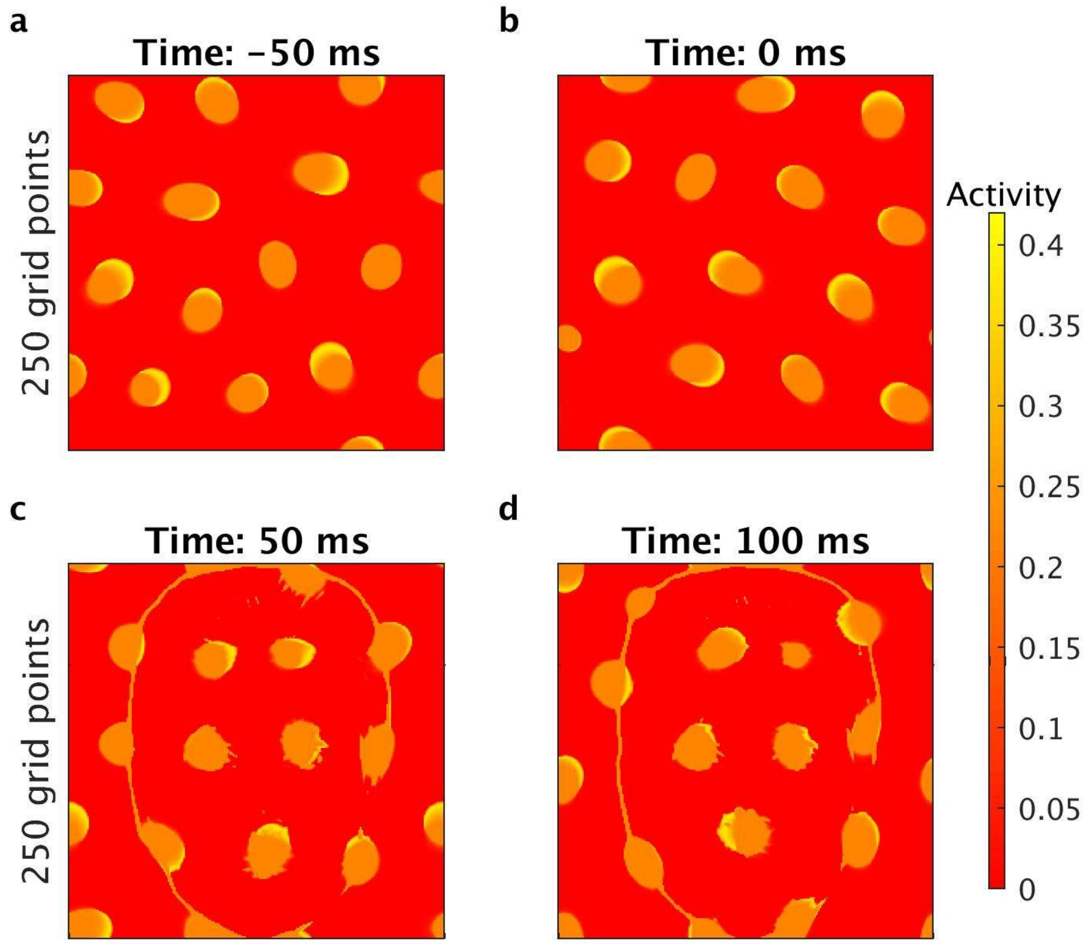

Supplementary Figure 17. In the firing rate model, spontaneous activity patterns are modulated by face images. a Snapshot of firing rate activity patterns generated by the firing rate model at 50 ms before the stimulus onset. **b** Same as **a** but at the stimulus onset moment. **c** Snapshot of firing rate activity patterns at 50 ms after the stimulus onset. The activity patterns are redistributed and modulated to the salient areas of the face image such as eyes and mouth. **d** Same as **c** but at 100 ms after the onset.

Supplementary Note 2

Analysis of the CMSA process. To gain a theoretical understanding of how and why the modulation process can account for the response property, we develop a stochastic model as mentioned in the main text. The model can be described as:

$$dX_t = \frac{nD}{X_t}dt + \sqrt{2D}dW_t \tag{5}$$

where W_t is standard Gaussian noise. The parameter D is the intensity of the noise, and n is the strength of the well. The corresponding Fokker-Planck equation for the probability density of the particle is

$$\frac{\partial p(x,t)}{\partial t} = -\frac{\partial}{\partial x}\left[\frac{nD}{x}p(x,t)\right] + D\frac{\partial^2 p(x,t)}{\partial x^2}\,. \tag{6}$$

In the following, it will be convenient to use the parameter $\nu = (1-n)/2$ instead of n. The first-exit time for this particle to leave $(0,\infty)$ was found by ref. [2] to have the density

$$f_{x_0}(T) = \frac{1}{\Gamma(\nu)}\left(\frac{x_0^2}{4D}\right)^{\nu} T^{-(\nu+1)} exp\left(-\frac{x_0^2}{4DT}\right)\,. \tag{7}$$

Broadly speaking, the response time is the first-exit time out of $(0,\infty)$ over a known prior for x_0, the initial position of the particle. We consider the general uniform prior for x_0 over (a,b). The response time density is then

$$\int_a^b f_{x_0}(T)dx_0 = \frac{1}{\Gamma(\nu)T}\int_a^b dx_0 \left(\frac{x_0^2}{4DT}\right)^{\nu} exp\left(-\frac{x_0^2}{4DT}\right) \tag{8}$$

$$= \frac{1}{\Gamma(\nu)T}\int_{\frac{a^2}{4DT}}^{\frac{b^2}{4DT}} dy\sqrt{\frac{DT}{y}}\,y^{\nu} exp(-y) \tag{9}$$

(change of variables with $y = x_0^2/(4DT)$, so $x_0 = \sqrt{4DTy}$ and $dx_0 = dy\sqrt{DT/y}$);

$$= \sqrt{\frac{D}{T}}\frac{1}{\Gamma(\nu)}\int_{\frac{a^2}{4DT}}^{\frac{b^2}{4DT}} dy\; y^{\nu-1/2}\mathrm{exp}(-y) \tag{10}$$

$$= \sqrt{\frac{D}{T}}\frac{\gamma\left(\nu+\frac{1}{2},\frac{b^2}{4DT}\right) - \gamma\left(\nu+\frac{1}{2},\frac{a^2}{4DT}\right)}{\Gamma(\nu)}, \tag{11}$$

where $\gamma(s,z)$ is the lower incomplete gamma function. Since $\gamma(s,0)=0$ and $\gamma(s,\infty)=\Gamma(s)$, the response time density becomes

$$\int_0^{\infty} f_{x_0}(T)dx_0 = \sqrt{\frac{D}{T}}\frac{\Gamma\left(\nu+\frac{1}{2}\right)}{\Gamma(\nu)}\,. \tag{12}$$

Response time for x_0 over $(0, b)$, where $b^2 \ll T$. Since $\gamma(s,z) \to z^s/s$ when $z \to 0$, the response time density becomes, for large T compared to b^2,

$$\int_0^b f_{x_0}(T)dx_0 \quad \to \sqrt{\frac{D}{T}}\frac{1}{\Gamma(\nu)}\frac{\left(\frac{b^2}{4DT}\right)^{\nu+1/2}}{\nu+1/2} \tag{13}$$

$$= \frac{\sqrt{D}}{\Gamma(\nu)(\nu+1/2)}\left(\frac{b^2}{4D}\right)^{\nu+1/2} T^{-(\nu+1)} \, . \tag{14}$$

Fig. 6 h shows the response time density when $D = 1/2$, $b = 1.5$, and $\nu = 1.5$ (corresponding to a power law exponent of -2.5).

Moments of the first-exit time. The mth moment of the first-exit time is

$$\langle T^m \rangle = \int_0^\infty T^m f_{x_0}(T)dT \tag{15}$$

$$= \frac{1}{\Gamma(\nu)}\int_0^\infty \left(\frac{x_0^2}{4DT}\right)^{\nu} T^{m-1}\, exp\left(-\frac{x_0^2}{4DT}\right) dT \tag{16}$$

$$= \frac{1}{\Gamma(\nu)}\int_0^\infty u^\nu \left(\frac{x_0^2}{4DT}\right)^{m-1} exp(-u)\left(-\frac{x_0^2}{4DT}du\right) \tag{17}$$

(change of variables with $u = x_0^2/(4DT)$, so $T = x_0^2/(4Du)$ and $dT = -du\; x_0^2/(4Du^2)$;

$$= \left(\frac{x_0^2}{4D}\right)^m \frac{1}{\Gamma(\nu)}\int_0^\infty u^{\nu-m-1} exp(-u)du \tag{18}$$

$$= \left(\frac{x_0^2}{4D}\right)^m \frac{\Gamma(\nu-m)}{\Gamma(\nu)}, \tag{19}$$

so the mean first-exit time varies as the square of the distance between the initial position of the particle and the center of the logarithmic potential well.

Supplementary References

1 Qi, Y. and Gong, P., Dynamic patterns in a two-dimensional neural field with refractoriness. *Phys. Rev. E* **92**, 22702 (2015).

2 Martin, E., Behn, U. and Germano, G., First-passage and first-exit times of a Bessel-like stochastic process. *Phys. Rev. E* **83**, 51115 (2011).
