# Supplementary material for: Computing by modulating spontaneous cortical activity patterns as a mechanism of active visual processing
Source: Nat Commun. 2019 Oct 29;10:4915. doi: 10.1038/s41467-019-12918-8 (PMC6820766; doi:10.1038/s41467-019-12918-8)
Supplement: Supplementary file 2 — Description of Additional Supplementary Files [file 41467_2019_12918_MOESM2_ESM.docx]

Description of Additional Supplementary Files

**Supplementary Movie 1.** The dynamics of membrane potentials at the critical point (Δ𝑊E = 0 𝜇S). The inter-frame time interval is 1 ms. The stimulus is added at 0 ms.

**Supplementary Movie 2.** The dynamics of membrane potentials at the patch-pattern-only state (Δ𝑊E = −0.1 𝜇S). The inter-frame time interval is 1 ms. The stimulus is added at 0 ms.

**Supplementary Movie 3.** The dynamics of membrane potentials at the regular-wave-only state (Δ𝑊E = 0.1 𝜇S). The inter-frame time interval is 1 ms. The stimulus is added at 0 ms.
